# 50 Years of Pollen Monitoring in Basel (Switzerland) Demonstrate the Influence of Climate Change on Airborne Pollen

**DOI:** 10.3389/falgy.2021.677159

**Published:** 2021-05-28

**Authors:** Regula Gehrig, Bernard Clot

**Affiliations:** Federal Office of Meteorology and Climatology MeteoSwiss, Zurich, Switzerland

**Keywords:** climate change, trends, long term data, pollen season definition, quality control

## Abstract

Climate change and human impact on vegetation modify the timing and the intensity of the pollen season. The 50 years of pollen monitoring in Basel, Switzerland provide a unique opportunity to study long-term changes in pollen data. Since 1969, pollen monitoring has been carried out in Basel with a Hirst-type pollen trap. Pollen season parameters for start dates, end dates and duration were calculated with different pollen season definitions, which are commonly used in aerobiology. Intensity was analyzed by the annual pollen integral (APIn), peak value and the number of days above specific thresholds. Linear trends were calculated with the non-parametric Mann Kendall method with a Theil-Sen linear trend slope. During the last 50 years, linear increase of the monthly mean temperatures in Basel was 0.95–1.95°C in the 3 winter months, 2–3.7°C in spring months and 2.75–3.85°C in summer months. Due to this temperature increase, the start dates of the pollen season for most of the spring pollen species have advanced, from 7 days for Poaceae to 29 days for *Taxus*/Cupressaceae. End dates of the pollen season depend on the chosen pollen season definition. Negative trends predominate, i.e., the pollen season mostly ends earlier. Trends in the length of the pollen season depend even more on the season definitions and results are contradictory and often not significant. The intensity of the pollen season of almost all tree pollen taxa increased significantly, while the Poaceae pollen season did not change and the pollen season of herbs decreased, except for Urticaceae pollen. Climate change has a particular impact on the pollen season, but the definitions used for the pollen season parameters are crucial for the calculation of the trends. The most stable results were achieved with threshold definitions that indicate regular occurrence above certain concentrations. Percentage definitions are not recommended for trend studies when the annual pollen integral changed significantly.

## Introduction

The number of published pollen season trend studies increased since the 1990s when changing temperatures and changing pollen season parameters became more and more obvious (1–3). At the beginning of this period, only few stations had data series long enough to study long term changes. Together with London, Leiden, Stockholm and Vienna, Basel was always one of these stations. Due to the growing number of pollen monitoring sites in Europe, and increasing length of the monitored period, it is nowadays possible to document changes in pollen season start, duration and intensity in a wide range of countries of Europe and North America (4, 5). Studies in the Southern hemisphere were less common, but a recent paper puts the focus on climate change and pollen aeroallergens in the Southern hemisphere (6).

One of the most influencing factor for changes in the pollen season characteristic is climate change (7). The changing climate alters the seasonality and the intensity of the pollen season and the distribution of allergenic plants. But also, allergenicity, dispersion and transport of pollen grains are influenced by climate parameters. Next to these, also other direct and indirect parameters of climate change like increasing CO_2_ concentrations and changes in air pollution can affect the plant physiology, the pollen grains and their allergenicity (8). All these makes allergy one of the major health effects resulting from a changing climate (8, 9). Changes in the pollen season characteristics, like increased intensity and longer duration, directly impacts the symptoms of allergic people or can lead to increased sensitization to specific pollen types (10, 11).

Many pollen season trend studies show changes toward earlier start dates. The advancing trend is clearer for spring, than for summer flowering taxa (5, 12, 13). Many studies show that phenological responses matched the warming pattern in Europe. Especially trends of spring and summer phases were strongly attributable to winter and spring warming (14–16). Non-significant trends or even delay of start dates may be caused by geographical location, plant types, regional changes in temperature or by short data series that lie within decadal fluctuations (5, 11, 17, 18). In contrast, trends for end dates and duration of the pollen season are more diverse. For tree pollen, many studies report no changes in the duration of the pollen season, while for herbaceous taxa more indications for a longer season exist (5, 13, 19–21).

Generally, the intensity of tree pollen taxa increased in Europe and North America, while the intensity of herbaceous taxa remained unchanged or decreased (4, 12). The intensity of the pollen season, the annual pollen integral (APIn), peak value or number of high pollen days, are very complex parameters with many influencing factors (4, 22, 23). Ziello et al. (24) did not find a correlation with temperature, but the increasing trends might be due to increasing CO_2_ concentrations. Effects of temperature and CO_2_ were shown in laboratory or free air carbon dioxide enrichment sites studies for Poaceae, *Ambrosia* and *Betula* (25–28) or between urban and rural areas with using natural temperature and CO_2_ gradients (29). A relation of the APIn and duration for total pollen with cumulative temperature and growing degree days could be shown in a global study (21). A number of studies showed the importance of weather conditions in the previous growing season for *Betula* APIn, next to a plant physiological biannual cycle and resource allocation mechanism (30–32). In forestry, an increase in mast year frequency has been observed (33, 34). Potential causes for this increase are rising temperatures during the vegetation period, a change in precipitation and water availability, a change in nitrogen deposition, increasing atmospheric CO_2_ levels or a general change in management and therefore an increase in available nutrients (35). Another very important factor for changes in the intensity of the pollen season is the human impact on vegetation cover and composition, i.e., increasing urbanization, area and management of urban green spaces, ornamental trees in towns, land use transformation and changes in agricultural practice (4, 36). In Helsinki it was found, that grass pollen concentrations followed well the land use patterns in the metropolitan area (37). The importance of the role of ornamental trees in town was demonstrated for *Platanus* pollen in Spanish cities, where a clear link between the growing numbers of plane trees and an increased APIn could be shown (38). The important influence of locally planted *Alnus x spaethii* trees on the start date and the intensity of the alder pollen season was shown for Buchs, Switzerland (39). The analyses of this human impact on vegetation are often hampered by a lack of data on long-term changes in land use and only very recent existence of tree inventories in towns.

In Switzerland, temperature has increased by about 2.0°C between 1864 and 2017 (40). Most of the warming takes place since the 1980s. The 1988–2017 summer average is by far the warmest 30-year summer average since the start of reliable reconstructions in 1685. Winter precipitation has increased by about 20–30% since 1864, although part of this apparent change may be due to natural variability. Sunshine duration shows a significant decline of −15 % between the 1950s and around 1980, followed by a significant increase of +20 % to the present day. No robust signals on long-term trends in the observational record are found for summer precipitation, droughts and wind speed (40).

Basel is one of the 14 pollen monitoring stations of the Swiss national pollen network. In Basel, a unique data set of 50 years allows for analyses of long-term changes in airborne pollen concentrations. Long term analysis over several decades can smooth out the influence of inter-annual and decadal variability of pollen data, which are also known from the global temperature behavior (41). Earlier studies of pollen trend analyses with data from the Swiss national pollen network were made for Basel (42, 43), Neuchâtel (44), for APIn (45) and for all 14 stations together (46). All these analyses cover shorter periods of up to 38 years.

There is no standard method to define the pollen season parameters of the start and end dates of the main pollen season (MPS). Jato et al. (47) failed to select one superior definition for delimiting the MPS and suggested that the appropriate one has to be selected depending on the goal of the study. The same was also recommended by Galán et al. and Pfaar et al. (48, 49). Bastl et al. (50) recommended the percentage definitions (e.g., starts at the day with 1% of the APIn and ends at the day of 95% of the APIn) for standard aerobiological routines such as pollen calendars and pollen season analysis, while the EAACI definitions (49) proved to be highly useful, when continuous exposure to a specific aeroallergen has to be assured, i.e., for clinical trials. Many commonly used pollen season definitions are implemented in the R-package AeRobiology in which also an overview is given of the source literature of these definitions (51).

The goal of this study is to calculate trends of pollen season parameters over 50 years for the most frequent pollen taxa, which we expect, will be more robust than with shorter data series. We will apply and compare several MPS definitions in order to recommend suitable ones for long term pollen trend studies. Quality control of the historic data will be addressed, especially the role of missing data and the influence of pollen trap relocations.

## Materials and Methods

### Location

Basel is located in the Rhine valley north of the Jura mountains, in the northwestern part of Switzerland. The town has 171'000 inhabitants. Basel is climatically favored and belongs to the stations with highest temperatures north of the Alps in Switzerland. The yearly mean temperature at the MeteoSwiss station Basel Binningen (316 m asl, distance of 2.3 km from the pollen trap) is 10.5°C and mean yearly precipitation sum is 842 mm (data for the normal period 1981–2010). All seasons are wet with seasonal precipitation sums of 158 mm in winter (DJF), 218 mm in spring (MAM), 257 mm in summer (JJA) and 210 mm in autumn (SON). The forests around Basel are mainly deciduous forests belonging to the categories of different types of beech and oak-hornbeam mixed forests (52), in which ash is always one of the dominant trees.

### Pollen Monitoring

The pollen trap of Basel belongs to the Swiss national pollen network which is run by the Federal Office of Meteorology and Climatology MeteoSwiss. Pollen monitoring has started in Basel on the 1th of March 1969 with a Hirst type pollen trap run by R. Leuschner in her PhD project (53). The pollen trap was located on the roof of a building at Petersplatz 11, 14.90 m above ground (47.559° N/7.583° E). The pollen trap was relocated in 1977 by 260 m to the roof of the Kantonsspital (University hospital) at 31.5 m above ground (47.562° N/7.584° E). Pollen sampling, sample preparation and pollen counting followed broadly the same methods during the whole 50-year period, which are very similar to the still valid ones (54). From 1969 until 2004 the adhesive and the mounting media were vaseline and Gelvatol, from 2005 until today it has been silicon oil and glycerine gelatine. The magnification of the microscope changed between 40, 50, and 60x. During the period of 1969–1993 the sampled area of the slide was alternately either 1 and 5 longitudinal transects (corresponds to 1.4%, respectively, 7.1% of the surface of the slide), while since 1994 always two longitudinal transects were read (this corresponds from 1994–2015 to 3.6% of the surface and since 2016 to 5.1%). The pollen monitoring and pollen slide reading was made by R. Leuschner from 1969 until 2004, since 1993 as one of the stations of the national pollen network of MeteoSwiss. Since 2005 the slides have been analyzed by the staff of the Swiss national pollen network of MeteoSwiss in Payerne. The long experience of pollen analysis by R. Leuschner guaranteed high data quality which was continued by MeteoSwiss. Quality control at MeteoSwiss is made by participation in European pollen quality control exercises (54, 55) and internal trainings.

### Selection of Pollen Taxa and Pollen Season Definitions

Out of the 48 pollen taxa analyzed by MeteoSwiss we selected the 13 most relevant taxa for allergies in Switzerland for this study. These are the tree pollen taxa *Corylus, Alnus, Fraxinus, Betula, Carpinus, Fagus, Quercus, Platanus* and the herbaceous taxa Poaceae*, Rumex, Plantago, Artemisia* and *Ambrosia*. In addition, other pollen taxa with an APIn of more than 500 pollen^*^day/m^3^ in the average of the 50-year period have been selected: the tree pollen taxa *Picea, Pinus, Populus, Taxus/*Cupressaceae (since 2009 these two taxa have been counted separately) and the herbaceous pollen type Urticaceae.

We applied eight different pollen season definitions (Table 1), knowing that they will show differing results for trend detections, as the recent study of 31 years of pollen trends in Switzerland shows (46). We applied two percentage methods, which are commonly used in aerobiological trend studies: “perc95” (2.5–97.5% of APIn) and “perc90” (5–95% of APIn) (56, 57). The other four definitions are based on thresholds. The clinical definitions of EAACI (49) were proposed for Central Europe with two different thresholds for *Betula* and Poaceae/*Artemisia*. Both definitions were applied to all taxa (“clin_bet,” “clin_poa”). The other threshold definitions are the first and the last day with ≥20 pollen/m^3^ and ≥30 pollen/m^3^ (“tr20,” “tr30”) and with 3 consecutive days with ≥20 pollen/m^3^ (“3d20”). We also considered the “moving average” definition proposed in the AeRobiology R package (51). This is a novel, not yet well-known method for pollen season determination. The pollen season parameters were calculated with the function “calculate_ps” of the AeRobiology package (51), but for the end of the pollen season with “moving average,” the last drop below the threshold of 5 pollen/m^3^ was used and not the first, like in the AeRobiology package. The “logistic” method (60) implemented in the AeRobiology package was also tested with the default program parameters. Due to several not possible, very early start date determinations, this method was not further used.

**Table 1:** List of the eight definitions used to determine start and end dates of the main pollen season (MPS).

**MPS Definition**	**Definition Type**	**Pollen Season Start**	**Pollen Season End**
clin_betclin_poa	Threshold	**Birch**: 1st day of 5 days with ≥10 pollen/m^3^ (out of 7 consecutive days) and sum of 5 days ≥100 pollen/m^3^**Grass**: 1st day of 5 days with ≥3 pollen/m^3^ (out of 7 consecutive days) and sum of 5 days ≥30 pollen/m^3^	**Birch**: Last day of 5 days with ≥10 pollen/m^3^ (out of 7 consecutive days) and sum of 5 days ≥100 pollen/m^3^ **Grass**: Last day of 5 days with ≥3 pollen/m^3^ (out of 7 consecutive days) and sum of 5 days ≥30 pollen/m^3^ (49)
perc90	Percentage	5% of APIn	95% of APIn (56)
perc95	Percentage	1% of APIn	95% of APIn (57)
tr20	Threshold	First day ≥20 pollen/m^3^	Last day ≥20 pollen/m^3^ (58)
tr30	Threshold	First day ≥30 pollen/m^3^	Last day ≥30 pollen/m^3^ (58)
3d20	Threshold	First day of 3 consecutive days of pollen with ≥20 pollen/m^3^	Last day of 3 consecutive days of pollen with ≥20 pollen/m^3^ (59)
moving	Threshold	First day of 11-day period when moving average pollen concentration is ≥5 pollen/m^3^	Last day of 11-day period when moving average pollen concentration is ≥5 pollen/m^3^ (51)

An APIn of more than 500 pollen^*^day m^3^ is considered high enough to calculate the pollen season parameters of start, end and duration in a reliable way. For lower APIn, the selected threshold definitions fail to determine start and end dates in many years (46). *Artemisia* and *Ambrosia* do not fulfill the criterion of an APIn of more than 500 pollen^*^day/m^3^ and for *Populus, Rumex, Plantago, Picea* APIn is in many years lower than 500 pollen^*^day/m^3^. Low APIn are not randomly distributed. Especially for the herbaceous taxa they occur more often toward the end of the data series. Therefore, for these taxa only trends of the pollen intensity parameters are calculated and not trends of start, end and duration of the pollen season with the 8 selected season definition. For the herbaceous taxa *Ambrosia, Artemisia, Plantago* and *Rumex*, lower threshold definitions were tested. A clinical definition with 5 days ≥ 1 pollen/m^3^ (out of 7 days) and sum of 5 days ≥10 pollen/m^3^ (“clin_low”), “3d1” and “5d1”: three and five consecutive days with at least 1 pollen/m^3^. The definitions “clin_poa” and “moving” could be applied for *Rumex* and *Plantago*, but not for *Ambrosia* and *Artemisia*.

### Data Analysis

#### Quality Control

Quality control of the data is required before calculating long term trends. Two aspects were controlled: first the influence of missing data on start dates, end dates and APIn and second whether the trap relocation in 1977 had an influence on pollen concentrations. Missing data was checked in a first step with the function “quality control” of the R-package AeRobiology (51) and the results were visually reviewed by expert judgement. In a second step the influence of missing data at the start dates of the pollen season were checked by phenological observations in the region of Basel with data from the Swiss phenology network of MeteoSwiss. This was especially the case for observation of *Corylus* flowering. This enabled to accept start dates of *Corylus* and *Alnus*, although pollen measurements only started in February or March in some years. A third step included the gap filling of pollen data with the function “interpollen” of the R-package AeRobiology (51) with the methods “lineal,” “movingmean,” “spline” and “tseries.” Pollen integrals of the gaps were calculated with the mean of all methods and a percentage loss of pollen was calculated. APIn values with <20% loss were accepted for being kept in the analysis. This number was chosen, because due to measurement errors of the Hirst type pollen trap a variability of up to 20% of APIn is expected (Adamov et al. On the measurement uncertainty of Hirst-type volumetric pollen and spore samplers, submitted). Gap filling was only used for quality checks, the following analysis were made without gap filled data series.

#### Breakpoint Detection

For the test of a possible influence of the pollen trap relocation or other changes in the applied methods, a breakpoint detection method was used for testing the APIn data series. If the relocation or method change had an influence on the data series, then breakpoints (or changepoints) in the data series of several pollen types should be visible. The breakpoint detection method of Penalized Maximal *F* test (PMF) was used with the online available software package RHtestsV4 (61–63). This software package can be used to detect multiple changepoints in climate data series. It is based on the penalized maximal *t* test and the penalized maximal *F* test (62), which are embedded in a recursive testing algorithm (61). The time series being tested may have no trend or a linear trend throughout the whole period of record. Breakpoints were analyzed for data series for each pollen taxa separately. Reference series are not available for break point detection. This break detection method was already applied for the Swiss phenological data series (64) and Swiss temperature series (65). Breakpoint detection with Bayesian methods was already applied for pollen data series of Switzerland, with the goal to analyze if trends are linear or if a model with breakpoints better describes the changes (45).

#### Pollen and Weather Data Analysis

Linear trends of the pollen season parameters APIn, peak value, number of high pollen days ≥50 pollen/m^3^, start date, end date and duration of the pollen season and the weather variables temperature and precipitation were calculated with Mann-Kendall trend test with Theil-Sen linear trend slope for the 50-year period 1969–2018. The weather variables are homogenized data series from the MeteoSwiss station Basel Binningen (316 m asl, distance of 2.3 km from the pollen trap). Spearman correlation analysis was made with monthly mean temperature and the start, end date, the duration and APIn of the pollen season for the different pollen season definitions for analyzing the influence of temperature on the pollen season parameters. For the presentation in the results chapter, the correlation coefficients of all pollen season definitions were averaged.

## Results

### Quality Control and Breakpoint Detection

Missing data mainly concerned the pollen season of *Corylus* and *Alnus*, for which the years 1969, 1974–1976, 1978, 1979, 1982 had to be omitted from all calculations of the pollen season parameters (Supplementary Figure 1) and for *Taxus/*Cupressaceae the years 1974–1976, 1979. For all other taxa, missing data did not influence the pollen season for more than 20% of APIn or for more than 2–4 days in pollen season start and end dates.

No significant breakpoints were detected in the APIn series in the years around the relocation of the pollen trap in 1977. This is an indication, that the relocation of the pollen trap did not influence the pollen concentrations, but without reference series it is not possible to prove that there really is no breakpoint. The significant breakpoints (*p*-value ≤ 0.05) for different pollen taxa are listed in Table 2 and Supplementary Figure 2. Taxa with no significant breakpoints are not mentioned. Second breakpoints in the data series (e.g., *Betula* in 1986) were not significant. The breakpoints for different pollen taxa are not synchronized in time. Deeper analysis would need to be made, to diagnose and explain the breakpoints in the years of 1999–2003 for tree pollen and Poaceae (*Betula, Taxus*/Cupressaceae, *Fraxinus, Pinus, Picea*) or during 1984–1987 for herbaceous taxa. The station history and changes in monitoring methods (e.g., change of the analyst, change of the counted area or the mounting media), do not provide evidence for these breaks.

**Table 2:** Significant breakpoints in the data series detected with the Penalized Maximal *F* test.

**Pollen taxa**	**Significant breaks**	**Pollen taxa**	**Significant breaks**
*Alnus*	1988	*Picea*	2003
*Taxus*/Cupressaceae	2000	Poaceae	2001
*Fraxinus*	2002	Urticaceae	1994
*Betula*	1999	*Plantago*	1984
*Quercus*	1995	*Rumex*	1986
*Pinus*	2002	*Ambrosia*	1987

### Trends in Pollen Data Series

#### Start Dates

**Figure 1:**
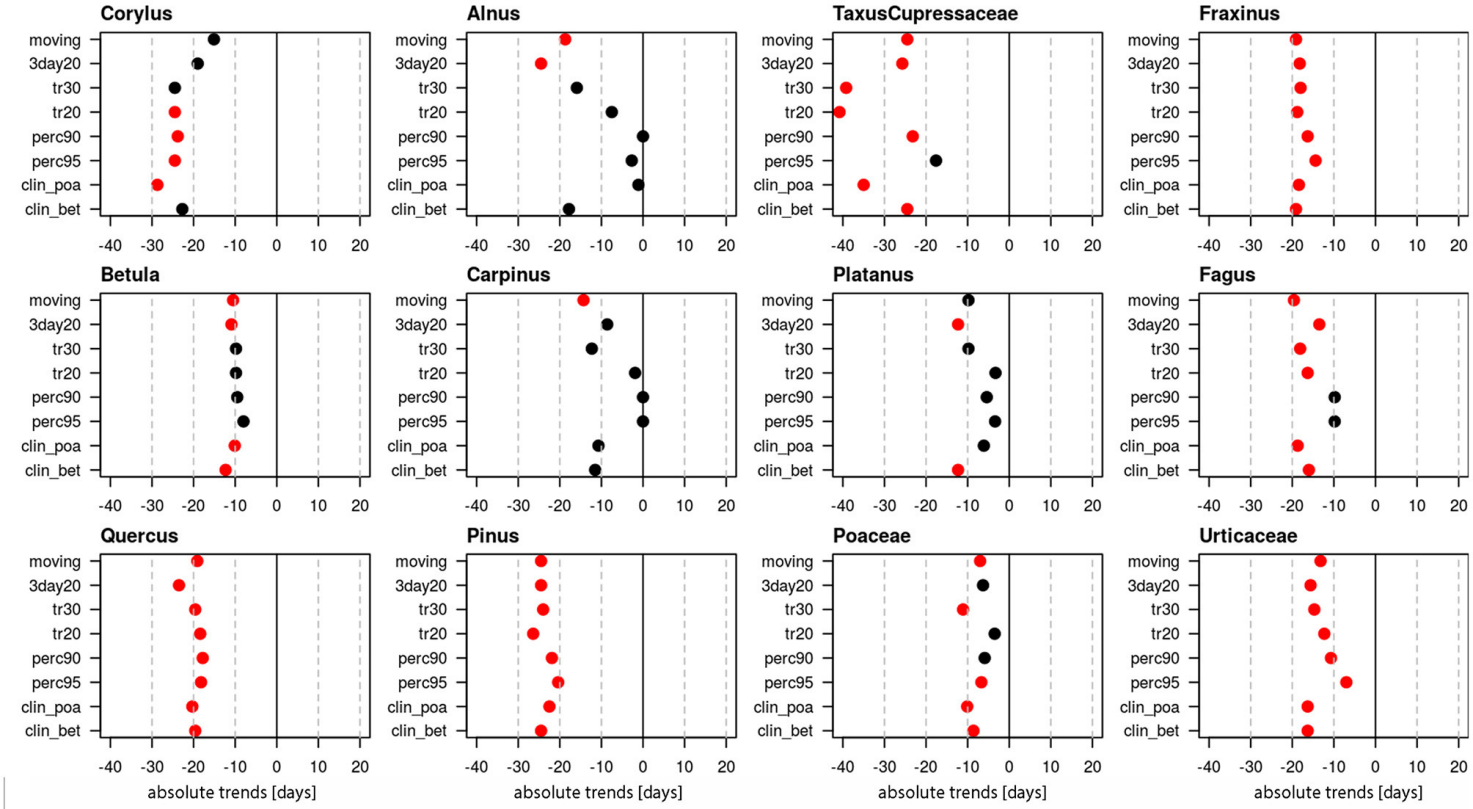
Absolute trends [days] for the 50-year period 1969–2018 of the start dates of the pollen season with eight different pollen season definitions. Red points show significant trends (*p*-value ≤ 0.05), black points show non-significant trends.

Linear trends of start dates were calculated for 12 pollen taxa (Figure 1, Supplementary Table 1), of which 10 are arboreal and two herbaceous taxa. Figure 1 provides the absolute trends in days for the period 1969–2018 for each of the 8 definitions. For all pollen taxa trends are negative, i.e., the pollen season starts earlier today. Very clear trends toward earlier start dates are present for *Taxus*/Cupressaceae (17.6–40.8 days), *Fraxinus* (14.4–19.1 days), *Fagus* (9.8–19.1 days), *Quercus* (17.8–23.5 days), *Pinus* (20.4–26.4 days) and Urticaceae (7–16.3 days). For these pollen taxa all or the majority of pollen season definitions show significant trends (*p* ≤ 0.05). For *Corylus* (15.1–28.7 days), *Betula* (9.8–12.3 days), Poaceae (3.5–11.1 days) only half of the definitions show significant changes, while for *Alnus, Carpinus* and *Platanus* the majority of the definitions do not show a significant advance.

**Figure 2:**
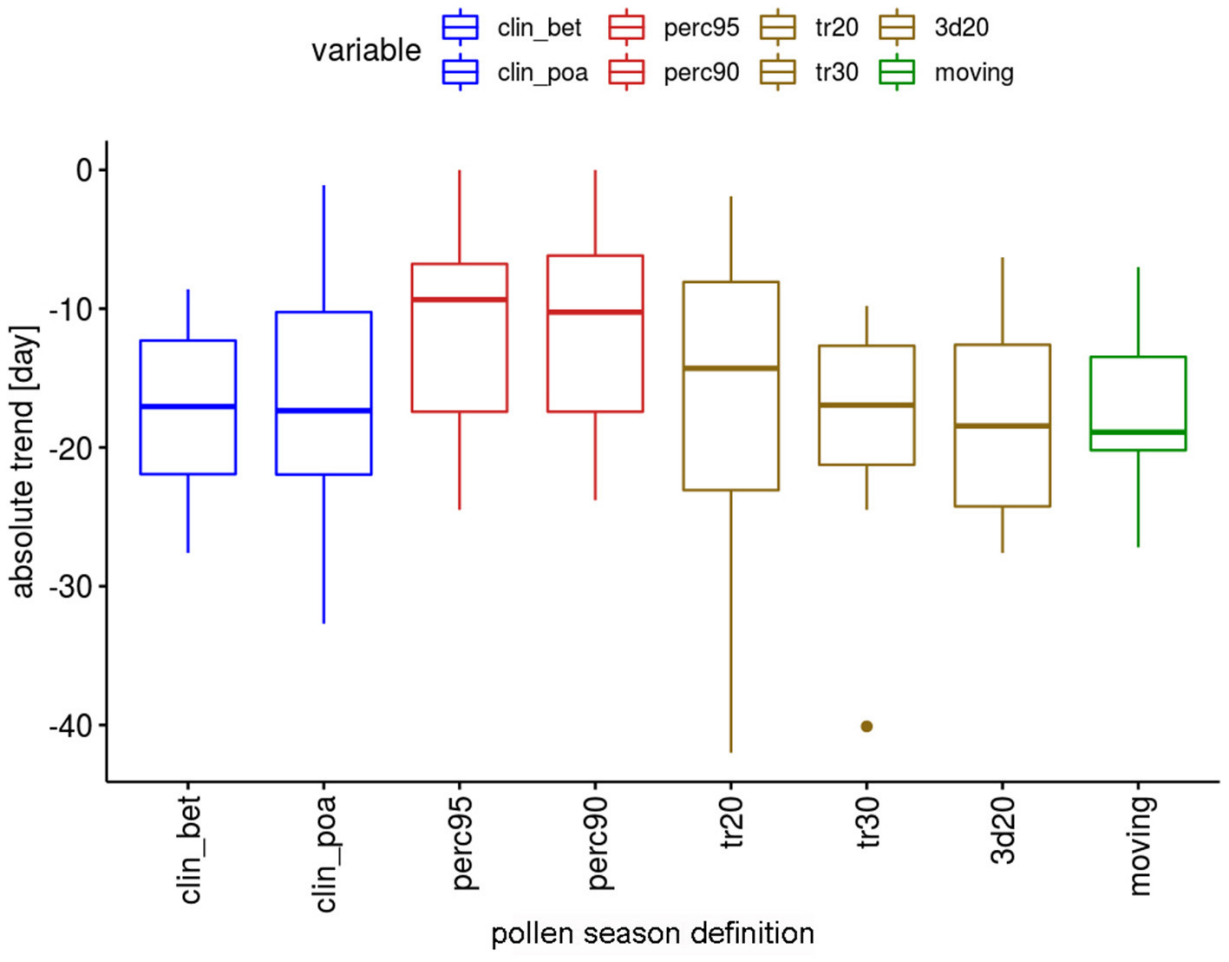
Range of absolute trends [days] for the 50-year period 1969–2018 of the start dates of the pollen season by definition for 12 pollen taxa.

The absolute trends differ depending on the definitions (Figure 2). For the two percentage definitions the median trends for the 12 pollen taxa are 8.9 or 10.3 days in the 50-year period. The threshold definitions result in larger trends with 15.9–17.4 days, with a very similar order of magnitude.

Trends for start, end and duration of the pollen season of *Ambrosia, Artemisia, Rumex* and *Plantago* with differing threshold definitions are presented in Supplementary Figure 3.

#### End Dates

**Figure 3:**
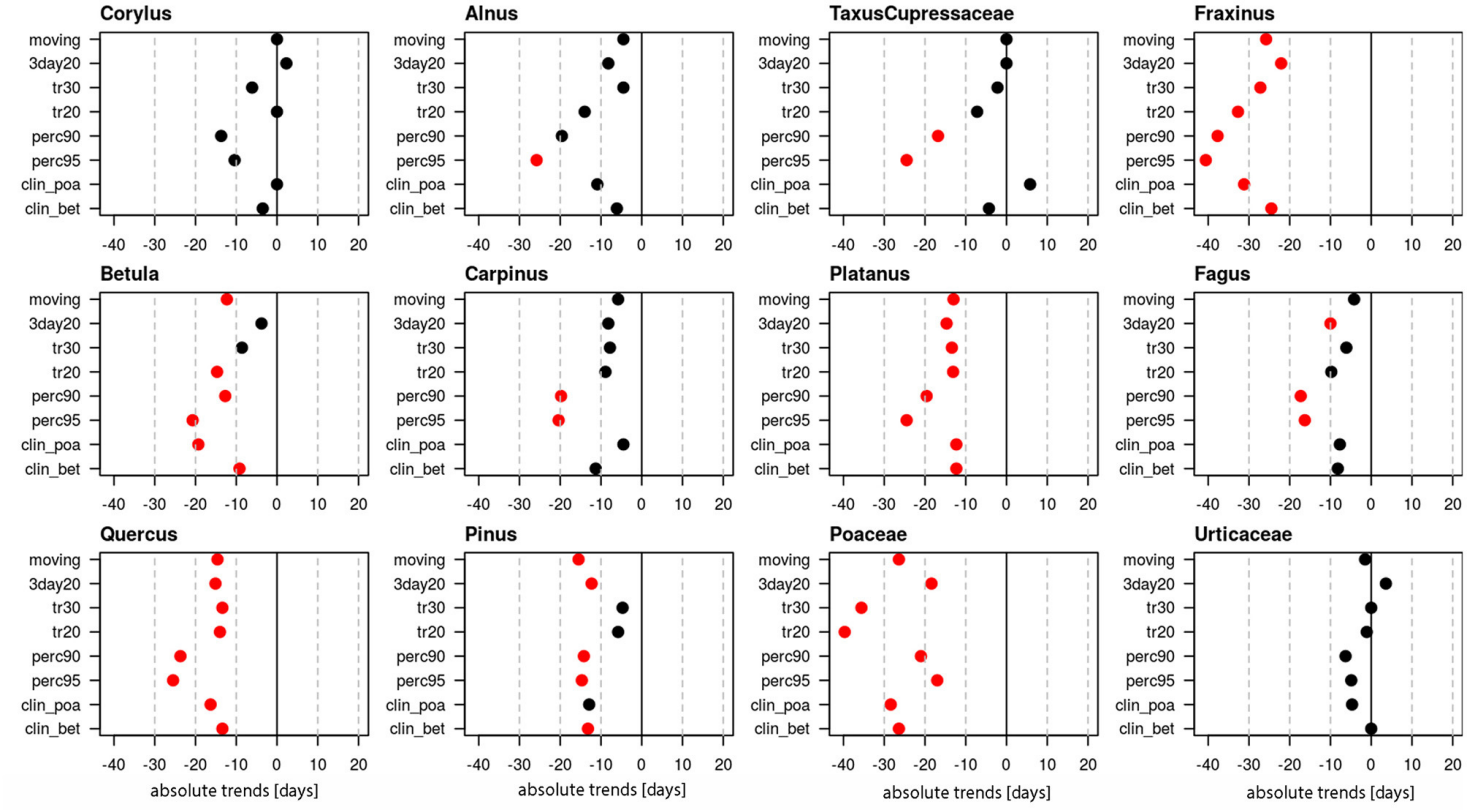
Absolute trends [days] for the 50-year period 1969–2018 of the end dates of the pollen season with eight different pollen season definitions. Red points show significant trends (*p*-value ≤ 0.05), black points show non-significant trends.

End dates of the pollen season generally have become earlier (Figure 3). Very clear trends for significant changes to earlier end dates for the majority of definitions are present for *Fraxinus* (24.5–40.6 days), *Betula* (9.2–20.7 days), *Platanus* (12.3–24.5 days), *Quercus* (13.4–25.5 days), *Pinus* (4.7–15.5 days) and Poaceae (17.0–39.7 days). Non-significant tendencies for earlier end dates are visible for *Alnus* (4.5–25.8 days), *Carpinus* (4.5–20.4 days) and *Fagus* (6.1–17.3 days). For *Corylus* and Urticaceae the pollen season end did not change significantly and for *Taxus*/Cupressaceae the trends are contradictory and only the percentage definitions show an earlier end of the pollen season.

**Figure 4:**
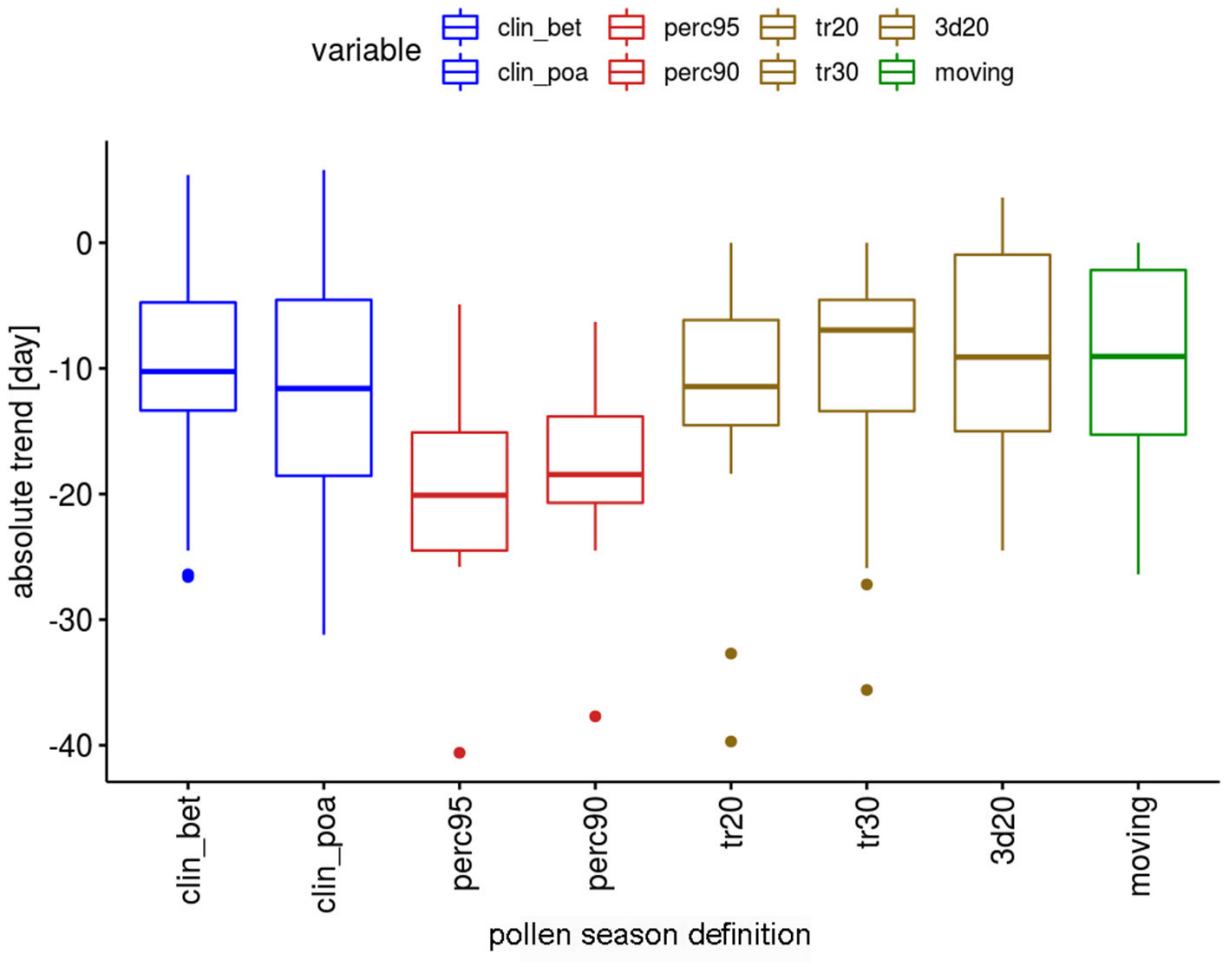
Range of absolute trends [days] for the 50-year period 1969–2018 of the end dates of the pollen season by definition for 12 pollen taxa.

The absolute trends are again dependent on the used definition of the pollen season (Figure 4). The most important advances of end dates are calculated with the percentage definitions, which have median values of advances of 18.5 or 20.6 days. The threshold definitions are in themselves rather homogeneous with median advances of 7–11.6 days.

#### Duration

**Figure 5:**
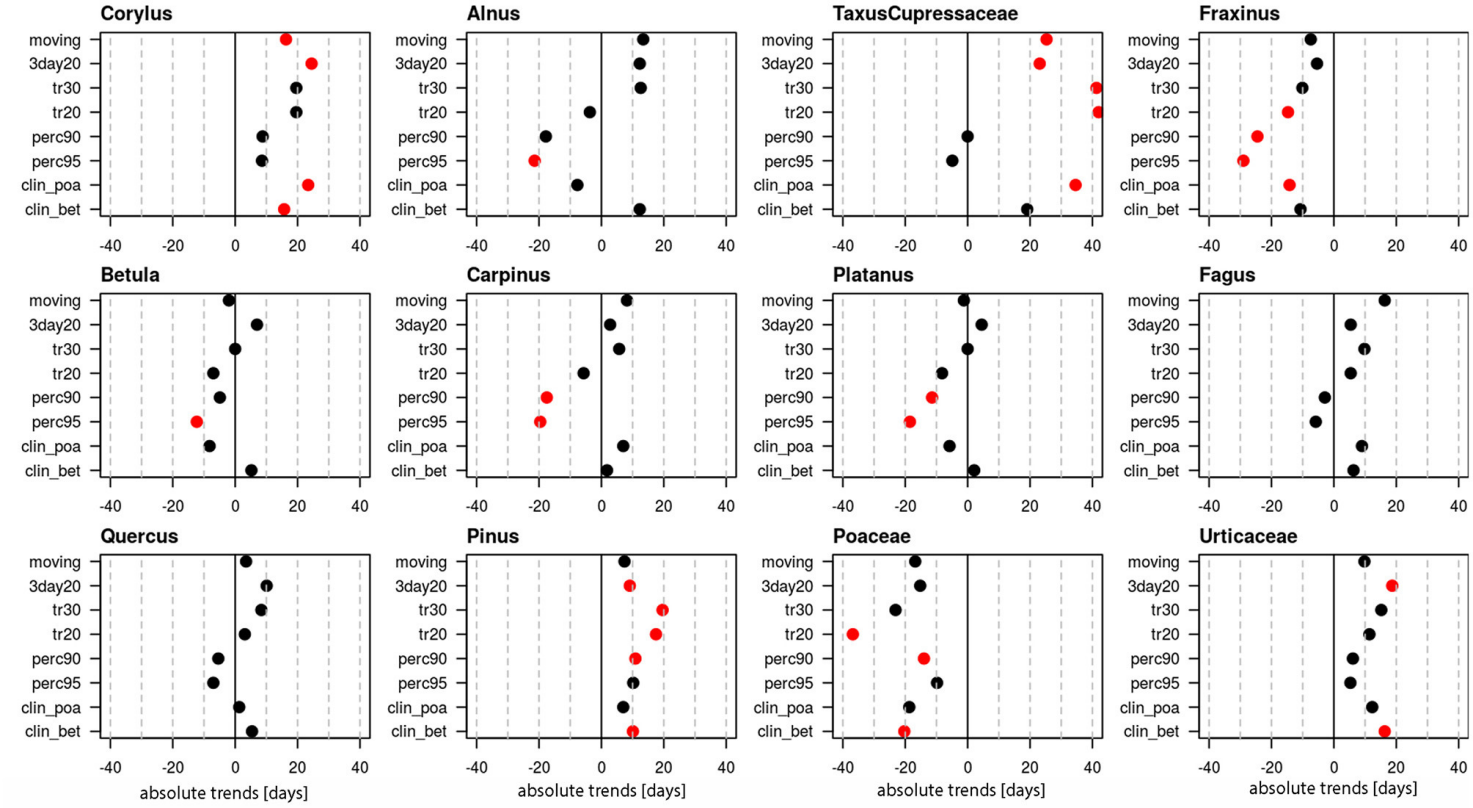
Absolute trends [days] for the 50-year period 1969–2018 of the duration of the pollen season with eight different pollen season definitions. Red points show significant trends (*p*-value ≤ 0.05), black points show non-significant trends.

Tendencies toward longer pollen seasons are present for *Corylus, Taxus*/Cupressaceae, *Pinus* and Urticaceae, while *Fraxinus* and Poaceae have a tendency toward a shorter pollen season (Figure 5). All these changes are significant just for single definitions. Only for *Pinus* und *Taxus*/Cupressaceae the majority, namely 5 out of 8 definitions show a prolongation. For all other pollen taxa, trends of duration are mostly contradictory for the applied definitions and the changes are not significant.

**Figure 6:**
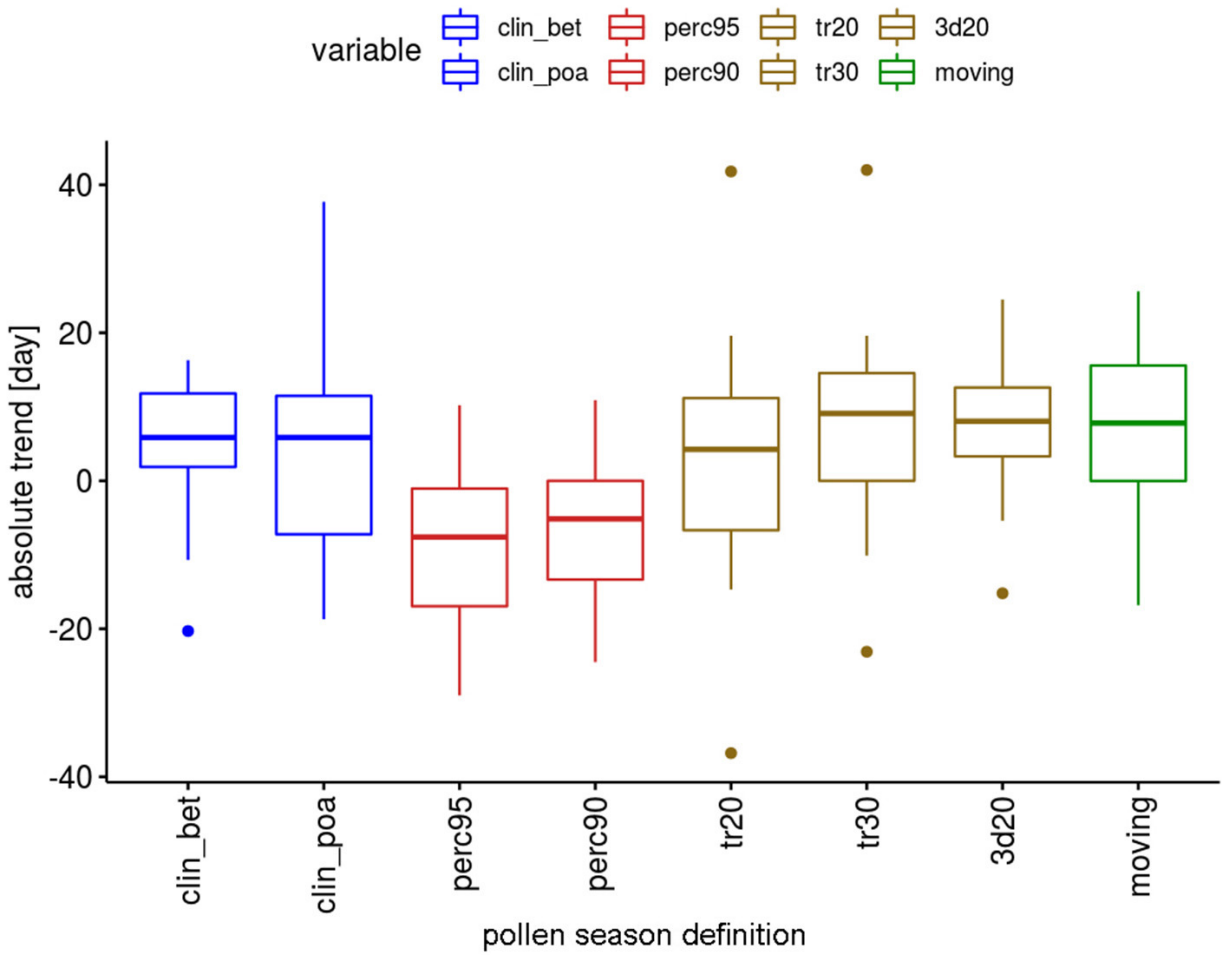
Range of absolute trends [days] for the 50-year period 1969–2018 of the duration of the pollen season by definition for 12 pollen taxa.

Although many trends are not significant, there are differences in the behavior of the definitions (Figure 6). For the percentage definitions more trends are significant, i.e., 5 out of 12 taxa. With the exception of *Pinus* (“perc90”) all these significant changes show a shortening of the pollen season. The threshold definitions provide significant changes for 2–4 taxa out of 12, with 72% of the significant changes for longer seasons.

The duration of the whole pollen season in Switzerland, calculated from the start of the *Corylus* season to the end of the Poaceae pollen season or to the end of the Urticaceae pollen season did not change significantly (Table 3). Tendencies for the duration differ for these two approaches for the whole pollen season either to a shortening or a prolongation. Although we see a clear advance of the start of the *Corylus* season, the Poaceae pollen season ends earlier. The Urticaceae season end did not change. The use of the allergenic species *Ambrosia* and *Artemisia* to delimit the pollen season would be preferable for allergy reasons, but due to an APIn frequently below 100 pollen^*^day/m^3^, the pollen season end can only be calculated with the percentage definitions. It is therefore not possible to limit the whole pollen season in Switzerland with these pollen taxa. Tests with much lower threshold definitions for *Plantago* show a longer total pollen season from *Corylus* flowering to the end of the *Plantago* pollen season, for the definitions of the last date of 3 consecutive days with 1 pollen/m^3^ and the last date with 5 consecutive days with 1 pollen/m^3^. Also, the “perc90” definition gives a prolongation of the pollen season, but attention has to be paid, because APIn of *Plantago* decreased significantly during the 50-years, so that this measure is not independent. The definition with slightly higher thresholds, “clin_poa” and “moving” show no significant trends.

**Table 3:** Absolute linear trends of the duration of the whole pollen season 1969–2018 from the *Corylus* flowering until the end of flowering of herbaceous taxa with different pollen season definitions.

	***Corylus*** **start to Poaceae end date**		***Corylus*** **start to Urticaceae end date**			***Corylus*** **start to** ***Plantago*** **end date**	
**Definition**	**Trend abs [days]**	***p*-value**	**Trend abs [days]**	***p*-value**	**Definition**	**Trend abs [days]**	***p*-value**
clin_bet	−14.2	0.429	20.7	0.129	*Corylus*: clin_bet *Plantago* clin_low	24.5	0.072
clin_poa	−4.7	0.722	17.8	0.061	*Corylus*: clin_poa *Plantago* clin_poa	6.4	0.738
perc95	0.0	0.925	13.5	0.129	*Corylus*: perc95 *Plantago*: perc95	22.3	0.060
perc90	−5.4	0.608	14.0	0.077	*Corylus*: perc90 *Plantago*: perc90	**27.2**	**0.004**
tr20	−24.5	0.084	12.9	0.221	*Corylus*: clin_poa *Plantago*: 3d1	**32.7**	**0.020**
tr30	−21.8	0.194	17.7	0.174	*Corylus*: clin_poa *Plantago*: 5d1	**30.9**	**0.044**
3d20	−12.3	0.369	17.9	0.208			
moving	−11.8	0.477	14.0	0.385	*Corylus*: moving *Plantago*: moving	−12.3	0.623

*On the left end dates calculated for Poaceae and Urticaceae with the eight pollen season definitions. On the right the trend for the whole season with end dates of Plantago with special, low threshold definitions and the definitions “clin_poa,” “moving” and the percentage definitions. Significant trends are marked in bold (p ≤ 0.05)*.

#### Intensity

**Figure 7:**
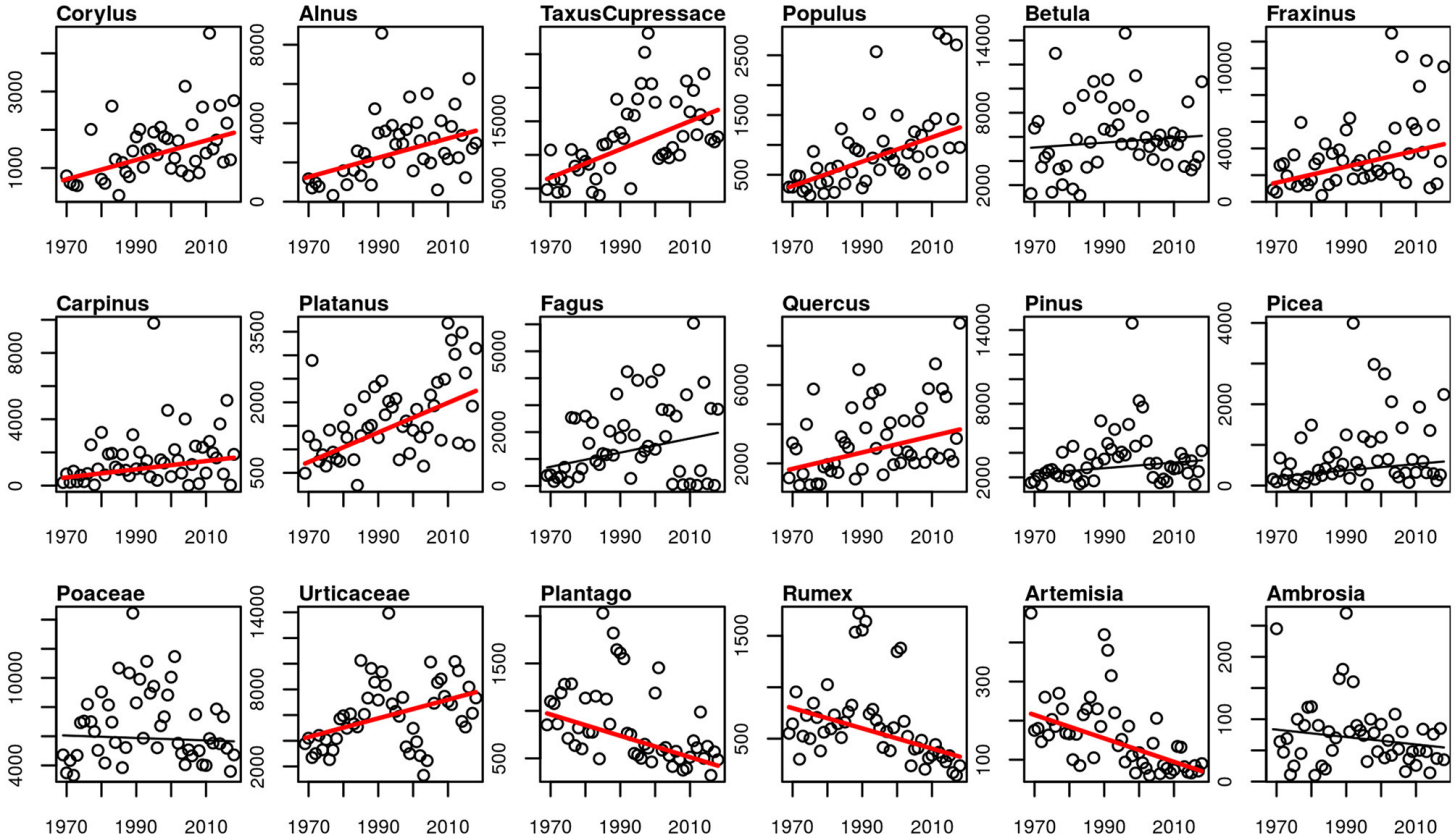
Linear trends of APIn for the 50-year period 1969–2018. Red trend lines show significant trends (*p*-value ≤ 0.05), black trend lines show non-significant trends.

The APIn of all studied arboreal pollen taxa increased, for most of the taxa significantly. Only for *Betula, Fagus, Pinus* and *Picea* the increase was not significant (*p*-values 0.498, 0.139, 0.105, 0.051). The only herbaceous taxa with an increase in the intensity of the pollen season is Urticaceae. Poaceae and *Ambrosia* APIn did not change, while the APIn of the rest of the herbaceous taxa decreased significantly (Figure 7). The same picture is also present for the peak value and the number of days above 50 pollen grains/m^3^ (Supplementary Figures 4, 5).

#### Weather Data and Pollen

Linear trends for the temperature and precipitation were calculated for the 50-year period in Basel. The yearly mean temperature increased by 2.4°C during the 50-year period 1969–2018 in Basel. The months with the strongest increases are April to August with a range of increase of 2.7–3.9°C (Table 4). The monthly temperatures of January to March did increase by 1–1.8°C, but the trend was not significant. Precipitation did not change in Basel, neither for monthly data nor for the whole year (Table 4).

**Table 4:** Linear trends of monthly mean temperature and monthly precipitation sum in Basel during the period 1969–2018.

**Month/Year**	**Temperature [** ^**°**^ **C]**		**Precipitation [mm]**	
	**Trend abs**	***p*-value**	**Trend abs**	***p*-value**
Jan	1.80	0.155	8.17	0.628
Feb	1.03	0.482	−0.26	0.967
Mar	1.81	0.059	−5.85	0.514
Apr	3.32	**<0.001**	2.97	0.861
May	2.90	**<0.001**	34.30	0.08
Jun	3.92	**<0.001**	−22.18	0.277
Jul	2.68	**0.001**	10.47	0.61
Aug	2.90	**<0.001**	8.52	0.682
Sep	1.63	**0.032**	12.25	0.32
Oct	2.45	**0.002**	23.46	0.216
Nov	1.68	**0.023**	−8.91	0.67
Dec	1.76	**0.041**	26.95	0.15
Year	2.4	**<0.001**	99.19	0.173

*Trend abs shows the absolute linear change of the parameter during the 50 years. Trends were calculated with Mann-Kendall trend test and Theil-Sen linear trend slope. Significant p-values are in bold*.

The most influencing period of temperature for start, end and duration of the pollen season was determined by correlation analysis with monthly mean data. Start dates correlate much better with temperature than end dates or season length. The pre-season period which influences the start dates are mainly the months in which mean flowering starts or 2–3 months before, depending on the pollen taxa. The correlation coefficients for the different season start definitions were averaged in the following description (Table 5). Highest correlations were found for *Betula* with March temperature (*r* = −0.838) and for *Fraxinus* with March temperature (*r* = −0.82) and mean February to March temperature (*r* = −0.787). The correlations for *Alnus* and *Corylus* were slightly lower and correlated best with January (*Corylus r* = −0.703 or January to February temperature (*Alnus r* = −0.720). A very high correlation could also be found for *Quercus* for March to April (*r* = −0.815). The correlation for the Poaceae pollen season was lower, and the most influencing pre-season period was either from January to May or February to May (*r* = −0.621, −0.610). No significant correlations were found with monthly temperatures of the autumn of the previous year, i.e., a chilling effect could not be shown.

**Table 5:** Correlation coefficients between temperature and start dates of the pollen season.

	**Jan**	**Mar**	**DecJan**	**JanFeb**	**JanMar**	**JanApr**	**JanMay**	**JanJun**	**FebMar**	**FebApr**	**FebMay**	**FebJun**	**MarApr**	**MarMay**	**MarJun**	**AprMay**	**AprJun**
*Alnus*	−0.534		−0.518	−0.720													
*Corylus*	−0.703		−0.658	−0.664													
*Tax*Cupr	−0.610		−0.591	−0.762	−0.749												
*Fraxinus*		−0.820			−0.705				−0.787								
*Betula*		−0.838			−0.691				−0.766								
*Carpinus*		−0.646			−0.574				−0.666								
*Platanus*		−0.645			−0.604	−0.576			−0.661	−0.662							
*Fagus*		−0.685							−0.629	−0.667			−0.637				
*Quercus*		−0.683				−0.646			−0.572	−0.717			−0.815				
*Pinus*							−0.661	−0.729		−0.685	−0.753	−0.801	−0.811	−0.816		−0.724	
Poaceae						−0.579	−0.621			−0.538	−0.610	−0.596		−0.566		−0.548	
Urticaceae														−0.516	−0.538	−0.551	−0.552

*The highest correlations with mean temperature of the monthly periods are shown. Correlation coefficients for all pollen season definitions were averaged. Correlations above |0.5| are significant with p < 0.001. Months without highly significant correlations are not listed (e.g., February)*.

The end of the pollen season correlates less well. For the arboreal pollen taxa, the temperature during the pollen season correlates mostly negatively with correlation coefficients of up to −0.785. For Poaceae, the correlation with April to July temperature is −0.533. For *Taxus/*Cupressaceae and Urticaceae correlations are not significant (Table 6).

**Table 6:** Correlation coefficients between temperature and end dates of the pollen season.

	**Feb**	**Mar**	**Jun**	**JanFeb**	**JanMar**	**JanApr**	**FebMar**	**FebApr**	**FebMai**	**FebJun**	**MarApr**	**MarMai**	**MarJun**	**AprMai**	**AprJun**	**AprJul**	**AprAug**
*Alnus*	−0.592	−0.687		−0.616	−0.776		−0.785										
*Corylus*	−0.593	−0.545		−0.629	−0.712		−0.724										
*Tax*Cupr																	
*Fraxinus*		−0.529				−0.538		−0.599			−0.727	−0.706					
*Betula*								−0.513			−0.641	−0.535					
*Carpinus*		−0.654			−0.534	−0.582	−0.526	−0.618			−0.667	−0.626					
*Fagus*														−0.462	−0.479		
*Quercus*									−0.527		−0.548	−0.607	−0.609	−0.592	−0.593		
*Platanus*		−0.580			−0.601	−0.667	−0.558	−0.682	−0.634		−0.681	−0.617					
*Pinus*												−0.504		−0.515			
Poaceae			−0.538												−0.521	−0.533	−0.516
Urticaceae																	

*The highest correlations with mean temperature of the monthly periods are shown. Correlation coefficients for all pollen season definitions were averaged. Correlations above |0.5| are significant with p < 0.001. Correlations for Taxus/Cupressaceae and Urticaceae are not significant. Months without highly significant correlations are not listed*.

The duration of the pollen season does not correlate significantly with temperature. Only the *Taxus*/Cupressaceae season length correlates positively with temperature from January to May (*r* = 0.516) (data not shown).

Correlations between APIn and temperature during pre-season or MPS exist, but most of them are below a correlation of *r* = |0.5| (Supplementary Table 4).

## Discussion

### Start Dates of MPS

Trends of an advance of the start dates of the pollen season are reported in most of the studies dealing with long-term pollen and climate change (5, 20, 21). In this study, all 12 analyzed pollen taxa show an advance of the start dates. The absolute trends differ clearly for the taxa and trends of the advance are either significant or not depending on the taxa or the start date definition. Clearest trends are observed for *Taxus*/Cupressaceae, *Fraxinus, Fagus, Quercus, Pinus* and Urticaceae. Only half of the season definitions show significant trends for *Corylus, Betula* and Poaceae, while for *Alnus, Carpinus* and *Platanus* trends are mostly not significant.

Generally, there are four basic factors governing the seasonal plant development: temperature (chilling and forcing), photoperiod and water availability (66). The main driver for the advancement of spring and early summer flowering, however, is temperature during the preseason period (19, 66–69). Preseason length for flowering in the Phenology Network of Switzerland was found to be between 50 and 80 days (67). We checked the preseason period by correlating monthly, 2 and 3 monthly mean temperature with pollen season start dates. Highest correlations were found with the temperature in the month of pollen season start or the 2 months or more seldom, 3 months before. Differences in the trends of start dates for pollen taxa can therefore be partly explained by different warming trends during the taxon specific preseason (Tables 4, 5). Preseason warming was stronger for taxa flowering later than mid-April (Table 4), like *Fagus, Quercus, Pinus* and Urticaceae. They all show clearer advancing trends than the taxa flowering earlier in the year. Exceptions from this are *Taxus*/Cupressaceae with a strong advance although the mean start date is the 12th of February and *Fraxinus* with a mean start date of 27th March. Another factor for the amount of trend in start dates is that the sensitivity to temperature is plant species specific (16). This may be the cause for the different trends of *Fraxinus* and *Betula*, although they are flowering almost simultaneously. *Fraxinus* is described as having less chilling requirements than *Betula* (70). Today the *Fraxinus* pollen season normally starts before the *Betula* season. But in the past, this was not the case. During many years from 1969 until the strong warming at the end of the 1980s, *Betula* was flowering earlier than *Fraxinus* (71).

Until today, winters in Switzerland are still cold enough, and a lack of chilling does not seem yet to play a role in the onset date of MPS and in spring plant phenology (67, 72). An example for this in the 50-year data series of Basel is the connection of *Betula* start dates with March temperature, the month with the highest correlation. The dependence is almost linear during the whole 50-year period. We do not see a change to an asymptotic behavior with higher March temperature, like it was observed for monitoring stations in UK (73) (Supplementary Figure 6). In future, inadequate chilling may become important, and the advancing trends will probably not continue (74). Photoperiod is a relevant factor for *Fagus, Ambrosia* and *Artemisia* and also grass species depend on chilling and long-day conditions (66, 70). This could probably be a reason why the advancing trend of *Fagus* is slightly smaller than the one of *Quercus* and that the advance of Poaceae is only about 10 days compared with the 20-day advance of *Quercus*.

The amount and significance of trends always depend on the studied period. Former studies in Switzerland showed much bigger trends for the advance of the *Betula* pollen season. In Neuchâtel a shift of 20 days for *Betula* start dates was found for the 21-year period 1979–1999 (trend 9.5 days/10 years) (44). In Basel the shift toward earlier *Betula* start dates was 15 days for the 38 years 1969–2006 (3.9 days/10 years) (43). During the 31-year period (1990–2020) *Betula* start dates remained unchanged in Switzerland (46). In our 50-year study we found an advance of 12 days for the “clin_bet” definition and 9.5 days for the “perc90” definition, which was also used by Clot (44), i.e., the 50-year trends in Basel are 2.4 days/10 years or 1.9 days/10 years. The main governing factor for *Betula* start dates is March temperature. Linear trends for these different periods for March temperature are for the 50-year period 1969–2018: 0.36°C/10 years, for the 38-year period 1969–2006: 0.63°C/10 years, for the 21-year period 1979–1999: 0.81°C/10 years and for 31-year period 1990–2020: −0.08°C/10 years. Not all of these March temperature changes are significant. The different changes in March temperature can explain the different trend amounts for studies covering different time periods. An additional factor may also be the use of different pollens season definitions (46).

### End Dates and Duration of MPS

The end dates of the pollen season of most of the taxa became earlier. Exceptions are *Corylus* and Urticaceae with not changing end dates. This means that for most of the taxa the pollen season has shifted as a whole toward earlier appearance and did not become longer. This is supported by mostly non-significant trends of the duration of the pollen season. For the arboreal pollen taxa, the temperature during the pollen season correlates mostly negatively with end dates, although not as clear as for start dates. The higher the temperature during the flowering period, the earlier the pollen season is ended. An earlier end of the arboreal taxa was also observed in Southern Germany (20) and in Belgium (19). The end of the Poaceae MPS advanced remarkably by 17–39.7 days and the pollen season has a tendency to become shorter, although only significant for 3 out of 8 definitions. The reason for these changes is most probably not the climate, but changes in land use and the frequency of grass cutting in agriculture. While in former times hay was made two times during summer, the grass is cut today a first time already before the flowering of the grasses and up to 5 times during summer (75). During the 50-year period in Basel a significant prolongation of the whole pollen season could not be observed in contradiction to many other aerobiological trend studies (20, 21, 76). The advance of the start of the *Corylus* flowering in January could not outweigh the earlier end of the grass pollen season or the unchanged end of the Urticaceae pollen season. This may be a local effect of the pollen trap in Basel, which is situated in the middle of the town and herbaceous pollen taxa may be underrepresented. When testing the whole pollen season with special low threshold definitions for the end of the *Plantago* pollen season, then an increase in the whole pollen season length of about 30 days is observed. The results of the *Plantago* pollen season show, that very low pollen concentrations have now a tendency to occur later in the year, although the trend is not significant. A similar effect can also be observed for Poaceae at very low concentrations. But this effect is not true for higher pollen concentrations, probably the ones relevant for allergies, because the end dates of the *Plantago* season with the definitions of “clin_poa” and “moving” became earlier over the 50-year period. Additional attention has to be paid when using such low pollen concentration thresholds at the end of the pollen season because of the high measurement uncertainty of pollen concentrations below 10 or 20 pollen/m^3^ (77) (Adamov et al. On the measurement uncertainty of Hirst-type volumetric pollen and spore samplers, submitted).

In the 31-year study with all 14 monitoring stations of the Swiss pollen network (46) a longer duration for *Corylus* and Urticaceae season was observed and the Poaceae pollen season became longer for four out of six pollen season definitions. These results demonstrates that during the most recent 30 years a prolongation of the pollen season can be observed in Switzerland. Next to the influence of the studied period, it also shows, that an analysis of several monitoring station together can give more stable results than just one station. Glick et al. (46) tested the pollen season definition used by Ziska et al. (21) (4th day of the initial 4 consecutive days of non-zero pollen collection). This definition showed clearly more significant prolongations of the pollen season than any other applied season definition, i.e., a similar behavior of low concentration definitions like in the present study.

### Intensity of the Pollen Season

Corresponding with many other aerobiological studies, we observed a clear trend toward more intense pollen seasons of most of the arboreal taxa (13, 19, 20, 24, 76). The exceptions are *Betula, Fagus, Pinus* and *Picea* with only non-significant tendencies for an increasing intensity. The pollen season intensity of herbaceous taxa *Plantago, Rumex* and *Artemisia* declined, while the season intensity of Poaceae and *Ambrosia* remained unchanged. Similar results for herbaceous pollen taxa were also found in a European wide study, with no changing or decreasing APIn (24). Poaceae and *Artemisia* intensity also declined in the 34-year study in Brussels (19), or were unchanged in Bavaria and Stockholm (13, 20). The exception is Urticaceae which show a significant increase of APIn, peak values and number of high pollen days. The reason for the increasing intensity of the Urticaceae pollen season in Basel is not yet known. Former studies in Switzerland show increasing *Corylus, Betula* and Poaceae APIn for Basel in the period 1969–1996 (42) and for the period 1969–2006 increasing *Betula* APIn and high pollen days (43). In Neuchâtel only four of 24 studied taxa APIn increased, *Alnus* and *Taxus*/Cupressaceae APIn and interestingly also *Artemisia* and *Ambrosia* (44). The 30-year study of 1985–2014 for Basel shows increased APIn for *Corylus* and decreased for *Betula* and Poaceae with Bayesian changepoint models (45). Due to a mostly high yearly and decadal variability of the APIn, the analysis concerning different time periods may give contradictory results in trends of the intensity.

In the data series of Basel only few relevant correlations between APIn and monthly mean temperatures during pre-season and MPS of the same year could be found. It is very difficult to separate the influence of temperature on pollen season intensity from all other influencing factors (4, 22, 23). Changes in land use, agriculture practices like mowing frequency and fertilizing, urban green spaces and planting of ornamental trees are influencing factors, which are very difficult to quantify (45, 66, 78, 79). Land use change, increase in built-up areas and decreasing agriculture area in Basel are most probably factors for the decrease of the APIn of herbaceous taxa and the not changing intensity of the Poaceae pollen season. From 1982–2014 the built-up area in Basel and in the surrounding district Arlesheim has increased by almost 10% from 6096 ha to 6623 ha, while the agriculture area has decreased from 3666 ha to 3131 ha (80). Land use changes were also described as reason for declining pollen intensities for grass pollen in England (81) or for ruderal taxa in Spain (36).

Water availability and precipitation during the preseason and the previous year are known to influence the intensity of the grass pollen season in the Mediterranean area (79, 82). Also, Rojo et al. (20) found indication, that the intensity of grass pollen seasons was governed by precipitation in spring in Bavaria, Germany. In Basel no correlation of the grass pollen season intensity parameters and precipitation could be found. It seems that until today, precipitation is not a limiting factor for grass pollen production in Switzerland. On the other hand, drought in summer can have an influence on the end of the grass pollen season. During the heat summer 2003 a very early end of the grass pollen season was observed in Switzerland (83) and also in Basel, the grass pollen season 2003 had the earliest end of the whole 50-year period (17th of July as a mean of all season definitions).

### Pollen Season Definitions

Pollen season definitions have been discussed in aerobiology since a long time and there is no solution for a unique definition, applicable for several scientific applications (47, 49, 50). It is recommended that the selection of the most suitable method be adapted to the main goal of the study (47, 48). Our research shows, that the pollen season definition has a strong influence on trends of the pollen season parameters and therefore also on studies analyzing influencing factors of climate change. The same results were also obtained for the 31-year trend study of pollen data of whole Switzerland (46). Differences between the definitions in start dates are quite small for taxa with an immediate, explosive increase of the daily pollen concentrations, like *Betula, Fraxinus* or *Quercus*. For these taxa, correlations between the different start date definitions are well-above 0.9. Differences are more important for pollen taxa with a slower increase of daily concentrations like Poaceae, Urticaceae or *Corylus* (Supplementary Tables 2, 3). Differences for the determination of end dates are even bigger, because the end of the pollen season normally fades out slowly in Basel. The EAACI definition for grass pollen (“clin_poa”) applied for Poaceae start dates correlates only with 0.43 to 0.71 (mean 0.534) with all other definitions, while the moving definition correlates with 0.60 to 0.82 (mean 0.744) (Supplementary Table 2). The “clin_poa” definition determines the earliest start dates for Poaceae, 4–18 days earlier than the other definitions. It has the advantage to determine pollen season when concentrations are still low but occurring regularly. For allergy studies, this definition will work well, because symptoms do occur as soon as pollen concentrations are low (49). Due to high measurement errors of the Hirst type pollen trap for low concentrations (Adamov et al. On the measurement uncertainty of Hirst-type volumetric pollen and spore samplers, submitted), this threshold based on low values can be reached sometimes by chance and is therefore less stable over a long time period. Climate change studies should refer on definitions with higher thresholds, to catch more stable start and end dates. The EAACI definitions with a higher threshold [“clin_bet,” or the cypress or olive definition (49)] fulfill this criterion. They have the additional advantage to demand regular presence of relevant pollen concentrations. Simple threshold definitions like the 1st day with 20 or 30 pollen/m^3^ may also occur partly at random and measurement errors can be important. The new approach of the moving average definition (“moving”) seems to be promising, but needs more tests with the selected thresholds for specific taxa (51). Unfortunately, the percentage definitions, which fulfill the criterion of being well-inside the pollen season, depend on APIn and are therefore not an independent measure. When API varies by year and when there is a long-term trend of APIn, these definitions contract or expand the pollen season independently from climate change (21, 47). This makes them not suitable for trend studies of start and end dates of the pollen season, although they have the advantage, that they can be applied also for pollen taxa with low APIn, for which threshold definitions are mostly not working. The percentage definitions clearly differ in trends for all three pollen season parameters. Due to the fact of increasing APIn of many arboreal taxa, the percentage definitions have lower trends for start dates, much more advances of the end dates and therefore frequently a more important trend to shorter pollen season than the threshold definitions. The opposite is true for herbaceous pollen types with decreasing APIn and the effect of increasing duration of the pollen season.

### Quality Control, Station Metadata, and Breakpoint Detection

Quality control, checking possible breakpoints in the data series, testing the influence of missing data and an accurate station history and station metadata are indispensable before calculating reliable trends and carry out climate change studies (84). A detailed station history with accurate description of the used method and instrument changes helps to detect and possibly correct changes in pollen concentration which are not due to climate change or other environmental parameters. Saar and Meltsov (85) described the importance of a passport of the sampling site by exactly recording vegetation and landscape around a pollen trap. The station history of Basel is not perfect and especially the influence of changes in the analyzed proportion of the tape or the possible changes in aspired air volume (86) cannot be reconstructed. We estimate that the error in the determination of the flow rate can affect historical data. Oteros et al. (86) do not recommend posterior correction of the flow rate with a static factor, because a considerable variation in the flow rate of different traps were found. We would like to motivate to better take care about metadata in aerobiology, especially when building new automated networks (87). Gap filling of missing data was approached by the R package AeRobiology (51). Gap filled data can be very useful for reducing the effect of pollen trap failures. Once pollen data will be gap filled, it will simplify the control of the data and allows to analyze complete measurement series. Clear criterions have to be established, on the size and proportion of gaps which can be filled. If possible, gap filling should not just be an interpolation of missing data but take into account neighboring stations and weather information. Then gap filling will be a helpful tool for improving pollen trend analysis and climate change studies. Break detection methods proved to be useful for analyzing influences of pollen trap relocation. Breakpoint detections will be more precise by using reference series, which was not possible in the case of Basel.

## Conclusion

We analyzed the 50-year pollen data series of Basel for trends in the pollen season parameters of start, end, duration and intensity of 12 pollen taxa. Such long term, multi decadal analysis can smooth out the influence of inter-annual and decadal variability of pollen data. Trends will be more robust than with shorter series. Most clear signals of influences of climate change were observed for start dates. For many of the taxa, start dates advanced significantly and correlated highly with pre-season temperature. Negative trends dominate for end dates of the pollen season, while the duration for most taxa did not change significantly. There was also no significant change in the whole pollen season from *Corylus* flowering to the end of the flowering of herbaceous taxa. The intensity of the pollen season of tree pollen taxa increased and decreased for herbaceous taxa or remained unchanged, with the exception of Urticaceae. We tested eight different pollen season definitions for delimiting MPS. Trend results of the definitions differed remarkably in the size or even direction of the trends. The most stable results were achieved with threshold definitions that indicate regular occurrence above certain concentrations. Percentage definitions are not recommended for trend studies in cases when the annual pollen integral changed significantly. Precise quality control of the data is required before calculating trends. Well-maintained meta data of the station are an important prerequisite for this for this.

## Data Availability Statement

The raw data supporting the conclusions of this article will be made available by the authors, without undue reservation.

## Author Contributions

RG made the conception and design of the study, performed the statistical analysis and wrote the first draft of the manuscript. RG and BC contributed to manuscript revision, read, and approved the submitted version.

## Conflict of Interest

The authors declare that the research was conducted in the absence of any commercial or financial relationships that could be construed as a potential conflict of interest.

## Abbreviations

APIn, Annual pollen integral: the sum of daily mean pollen concentrations [pollen^*^day/m^3^]
EAACI: European Academy of Allergy and Clinical Immunology
MPS: Main pollen season.

## References

[1] Emberlin J Savage M Jones S. Annual variations in grass pollen seasons in London 1961–1990: trends and forecast models. Clin Exp Allergy. (1993) 23:911–8. 10.1111/j.1365-2222.1993.tb00275.x 10779278

[2] Spieksma FTM Emberlin JC Hjelmroos M Jäger S Leuschner RM. Atmospheric birch (*Betula*) pollen in Europe: trends and fluctuations in annual quantities and the starting dates of the seasons. Grana. (1995) 34:51–7. 10.1080/00173139509429033

[3] Jäger S Nilsson S Berggren B Pessi AM Helander M Ramfjord H. Trends of some airborne tree pollen in the Nordic countries and Austria, 1980-1993. Grana. (1996) 35:171–8. 10.1080/00173139609429078

[4] Menzel A Jochner S. Impacts of climate change on aeroallergen production and atmospheric concentration. In: Beggs PJ, editor. Impacts of Climate Change on Allergens and Allergic Diseases. Cambridge, UK: Cambridge University Press (2016). p. 10–28. 10.1017/CBO9781107272859.003

[5] Ziska LH. Impacts of climate change on allergen seasonality. In: Beggs PJ, editor. Impacts of Climate Change on Allergens and Allergic Diseases. Cambridge, MA: Cambridge University Press (2016). p. 92–112. 10.1017/CBO9781107272859.007

[6] Davies JM Berman D Beggs PJ Ramón GD Peter J Katelaris CH. Global climate change and pollen aeroallergens: a Southern hemisphere perspective. Immunol Allergy Clin North Am. (2021) 41:1–16. 10.1016/j.iac.2020.09.002 33228867

[7] Beggs PJ. Impacts of Climate Change on Allergens and Allergic Diseases. Cambridge, MA: Cambridge University Press (2016). p. 193. 10.1017/CBO9781107272859

[8] D'Amato G Chong-Neto HJ Monge Ortega OP Vitale C Ansotegui I Rosario N. The effects of climate change on respiratory allergy and asthma induced by pollen and mold allergens. Allergy. (2020) 75:2219–28. 10.1111/all.14476 32589303

[9] EASAC. The Imperative of Climate Action to Protect Human Health in Europe. (2019). Available online at: https://easac.eu/fileadmin/PDF_s/reports_statements/Climate_Change_and_Health/EASAC_Report_No_38_Climate_Change_and_Health.pdf (accessed February 18, 2021).

[10] Ariano R Canonica GW Passalacqua G. Possible role of climate changes in variations in pollen seasons and allergic sensitizations during 27 years. Ann Allergy Asthma Immunol. (2010) 104:215–22. 10.1016/j.anai.2009.12.005 20377111

[11] Katelaris CH Beggs PJ. Climate change: allergens and allergic diseases. Intern Med J. (2018) 48:129–34. 10.1111/imj.13699 29415354

[12] Zhang Y Bielory L Mi Z Cai T Robock A Georgopoulos P. Allergenic pollen season variations in the past two decades under changing climate in the United States. Glob Change Biol. (2015) 21:1581–9. 10.1111/gcb.12755 25266307 PMC4356643

[13] Lind T Ekebom A Alm Kübler K Östensson P Bellander T Lõhmus M. Pollen Season Trends (1973-2013) in Stockholm Area, Sweden. PLoS ONE. (2016) 11:e0. 10.1371/journal.pone.0166887 27898718 PMC5127655

[14] Menzel A Sparks TH Estrella N Koch E Aasa A Ahas R. European phenological response to climate change matches the warming pattern. Glob Change Biol. (2006) 12:1–8. 10.1111/j.1365-2486.2006.01193.x

[15] Parmesan C. Influences of species, latitudes and methodologies on estimates of phenological response to global warming. Glob Change Biol. (2007) 13:1860–72. 10.1111/j.1365-2486.2007.01404.x

[16] Menzel A Yuan Y Matiu M Sparks T Scheifinger H Gehrig R. Climate change fingerprints in recent European plant phenology. Glob Change Biol. (2020) 26:2599–612. 10.1111/gcb.15000 31950538

[17] Levetin E de Water P. Changing pollen types/concentrations/distribution in the United States: Fact or fiction? Curr Allergy Asthma Rep. (2008) 8:418–24. 10.1007/s11882-008-0081-z 18682110

[18] Ugolotti M Pasquarella C Vitali P Smith M Albertini R. Characteristics and trends of selected pollen seasons recorded in Parma (Northern Italy) from 1994 to 2011. Aerobiologia. (2015) 31:341–52. 10.1007/s10453-015-9368-4

[19] Hoebeke L Bruffaerts N Verstraeten C Delcloo A De Smedt T Packeu A. Thirty-four years of pollen monitoring: an evaluation of the temporal variation of pollen seasons in Belgium. Aerobiologia. (2018) 34:139–55. 10.1007/s10453-017-9503-5

[20] Rojo J Picornell A Oteros J Werchan M Werchan B Bergmann K-C. Consequences of climate change on airborne pollen in Bavaria, Central Europe. Reg Environ Change. (2021) 21:9. 10.1007/s10113-020-01729-z

[21] Ziska LH Makra L Harry SK Bruffaerts N Hendrickx M Coates F. Temperature-related changes in airborne allergenic pollen abundance and seasonality across the northern hemisphere: a retrospective data analysis. Lancet Planet Heal. (2019) 3:e124–31. 10.1016/S2542-5196(19)30015-4 30904111

[22] Clot B Gehrig R Pauling A Pietragalla B. The wind of change: effects of climate change on airborne pollen concentrations. Alergol Immunol. (2012) 9:139–40.

[23] Damialis A Traidl-Hoffmann C Treudler R. Climate change and pollen allergies. In: Marselle MR Stadler J Korn H Irvine KN Bonn A, editors. Biodiversity and Health in the Face of Climate Change. Cham: Springer International Publishing (2019). p. 47–66. 10.1007/978-3-030-02318-8_3

[24] Ziello C Sparks TH Estrella N Belmonte J Bergmann KC Bucher E. Changes to Airborne pollen counts across Europe. PLoS ONE. (2012) 7:e34076. 10.1371/journal.pone.0034076 22514618 PMC3325983

[25] Ziska LH Caulfield FA. Rising CO2 and pollen production of common ragweed (*Ambrosia artemisiifolia* L.), a known allergy-inducing species: implications for public health. Aust J Plant Physiol. (2000) 27:893–8. 10.1071/PP00032

[26] Rogers CA Wayne PM Macklin EA Muilenberg ML Wagner CJ Epstein PR. Interaction of the onset of spring and elevated atmospheric CO2 on Ragweed (*Ambrosia artemisiifolia* L.) pollen production. Environ Health Perspect. (2006) 114:865–9. 10.1289/ehp.8549 16759986 PMC1480488

[27] Albertine JM Manning WJ DaCosta M Stinson KA Muilenberg ML Rogers CA. Projected carbon dioxide to increase grass pollen and allergen exposure despite higher ozone levels. PLoS ONE. (2014) 9:e111712. 10.1371/journal.pone.0111712 25372614 PMC4221106

[28] Darbah JNT Kubiske ME Nelson N Oksanen E Vapaavuori E Karnosky DF. Effects of decadal exposure to interacting elevated CO2 and/or O3 on paper birch (*Betula papyrifera*) reproduction. Environ Pollut. (2008) 155:446–52. 10.1016/j.envpol.2008.01.033 18355950

[29] Ziska LH Gebhard DE Frenz DA Faulkner S Singer BD Straka JG. Cities as harbingers of climate change: common ragweed, urbanization, and public health. J Allergy Clin Immunol. (2003) 111:290–5. 10.1067/mai.2003.53 12589347

[30] Dahl A Strandhede S-O. Predicting the intensity of birch pollen season. Aerobiologia. (1996) 12:97–106. 10.1007/BF02248133

[31] Rasmussen A. The effects of climate change on the birch pollen season in Denmark. Aerobiologia. (2002) 18:253–65. 10.1023/A:1021321615254

[32] Ranta H Oksanen A Hokkanen T Bondestam K Heino S. Masting by *Betula*-species; applying the resource budget model to north European data sets. Int J Biometeorol. (2005) 49:146–51. 10.1007/s00484-004-0228-0 15340828

[33] Övergaard R Gemmel P Karlsson M. Effects of weather conditions on mast year frequency in beech (*Fagus sylvatica* L.) in Sweden. Forestry. (2007) 80:555–65. 10.1093/forestry/cpm020

[34] Paar U Guckland A Dammann I Albrecht M Eichhorn J. Häufigkeit und Intensität der Fruktifikation der Buche. AFZ-Der Wald. (2011) 6:26–9.

[35] Nussbaumer A Waldner P Etzold S Gessler A Benham S Thomsen IM. Patterns of mast fruiting of common beech, sessile and common oak, Norway spruce and Scots pine in Central and Northern Europe. For Ecol Manage. (2016) 363:237–51. 10.1016/j.foreco.2015.12.033

[36] García-Mozo H Oteros JA Galán C. Impact of land cover changes and climate on the main airborne pollen types in Southern Spain. Sci Total Environ. (2016) 548:221–8. 10.1016/j.scitotenv.2016.01.005 26802350

[37] Hjort J Hugg TT Antikainen H Rusanen J Sofiev M Kukkonen J. Fine-Scale exposure to allergenic pollen in the Urban environment: evaluation of land use regression approach. Environ Health Perspect. (2016) 124:619–26. 10.1289/ehp.1509761 26452296 PMC4858385

[38] Lara B Rojo J Fernández-González F González-García-Saavedra A Serrano-Bravo MD Pérez-Badia R. Impact of plane tree abundance on temporal and spatial variations in pollen concentration. Forests. (2020) 11:817. 10.3390/f11080817

[39] Gehrig R Gassner M Schmid-Grendelmeier P. *Alnus* × *spaethii* pollen can cause allergies already at Christmas. Aerobiologia. (2015) 31:239–47. 10.1007/s10453-014-9360-4

[40] CH2018. CH2018 – Climate Scenarios for Switzerland. Zurich: Technical Report, National Centre for Climate Services (2018). p. 271.

[41] Muller RA Curry J Groom D Jacobsen R Perlmutter S Rohde R. Decadal variations in the global atmospheric land temperatures. J Geophys Res Atmos. (2013) 118:5280–6. 10.1002/jgrd.5045831012512

[42] Frei T. The effects of climate change in Switzerland 1969–1996 on airborne pollen quantities from hazel, birch and grass. Grana. (1998) 37:172–9. 10.1080/00173139809362662

[43] Frei T Gassner E. Climate change and its impact on birch pollen quantities and the start of the pollen season an example from Switzerland for the period 1969–2006. Int J Biometeorol. (2008) 52:667–74. 10.1007/s00484-008-0159-2 18481116

[44] Clot B. Trends in airborne pollen: an overview of 21 years of data in Neuchâtel (Switzerland). Aerobiologia. (2003) 19:227–34. 10.1023/B:AERO.0000006572.53105.17

[45] Jochner-Oette S Menzel A Gehrig R Clot B. Decrease or increase? Temporal changes in pollen concentrations assessed by Bayesian statistics. Aerobiologia. (2019) 35:153–63. 10.1007/s10453-018-9547-1

[46] Glick S Gehrig R Eeftens M. Multi-decade changes in pollen season onset, duration, and intensity: a concern for public health? Sci Total Environ. (2021) 781:146382. 10.1016/j.scitotenv.2021.146382 33812098 PMC8182784

[47] Jato V Rodríguez-Rajo FJ Alcázar P De Nuntiis P Galán C Mandrioli P. May the definition of pollen season influence aerobiological results? Aerobiologia. (2006) 22:13–25. 10.1007/s10453-005-9011-x

[48] Galán C Ariatti A Bonini M Clot B Crouzy B Dahl A. Recommended terminology for aerobiological studies. Aerobiologia. (2017) 33:293–5. 10.1007/s10453-017-9496-0

[49] Pfaar O Bastl K Berger U Buters J Calderon MA Clot B. Defining pollen exposure times for clinical trials of allergen immunotherapy for pollen-induced rhinoconjunctivitis - an EAACI position paper. Allergy. (2017) 72:713–22. 10.1111/all.13092 27874202

[50] Bastl K Kmenta M Berger UE. Defining pollen seasons: background and recommendations. Curr Allergy Asthma Rep. (2018) 18:73. 10.1007/s11882-018-0829-z 30374908 PMC6209030

[51] Rojo J Picornell A Oteros J. AeRobiology: the computational tool for biological data in the air. Methods Ecol Evol. (2019) 10:1371–6. 10.1111/2041-210X.13203

[52] Ellenberg H Klötzli F. Waldgesellschaften und Waldstandorte der Schweiz. Mitteilungen Schweiz Anst Forstl Versuchsw. (1972) 48:589–930.

[53] Leuschner RM. Luftpollenbestimmung in Basel Während der Jahre 1969 und 1970. Basel: Universität Basel (1974).

[54] Galán C Smith M Thibaudon M Frenguelli G Oteros J Gehrig R. Pollen monitoring: minimum requirements and reproducibility of analysis. Aerobiologia. (2014) 30:385–95. 10.1007/s10453-014-9335-5

[55] Sikoparija B Galán C Smith M EAS QC Working Group. Pollen-monitoring: between analyst proficiency testing. Aerobiologia. (2017) 33:191–9. 10.1007/s10453-016-9461-3

[56] Nilsson S Persson S. Tree pollen spectra in the stockholm region (Sweden), 1973-1980. Grana. (1981) 20:179–82. 10.1080/00173138109427661

[57] Andersen TB. A model to predict the beginning of the pollen season. Grana. (1991) 30:269–75. 10.1080/00173139109427810

[58] Giorato M Lorenzoni F Bordin A De Biasi G Gemignani C Schiappoli M. Airborne allergenic pollens in Padua: 1991-1996. Aerobiologia. (2000) 16:453–4. 10.1023/A:1026570709638

[59] Galán C García-Mozo H Cariñanos P Alcãzar P Domínguez-Vilches E. The role of temperature in the onset of the Olea europaea L. pollen season in southwestern Spain. Int J Biometeorol. (2001) 45:8–12. 10.1007/s004840100089 11411416

[60] Ribeiro H Cunha M Abreu I. Definition of main pollen season using a logistic model. Ann Agric Environ Med. (2007) 14:259–64. 18247462

[61] Wang XL. Accounting for autocorrelation in detecting mean shifts in climate data series using the penalized maximal t or F test. J Appl Meteorol Climatol. (2008) 47:2423–44. 10.1175/2008JAMC1741.1

[62] Wang XL. Penalized maximal F test for detecting undocumented mean shift without trend change. J Atmos Ocean Technol. (2008) 25:368–84. 10.1175/2007JTECHA982.1

[63] Wang XL Feng Y. RHtests V4 User Manual. Climate Research Division, Atmospheric Science and Technology Directorate, Science and Technology Branch, Environment Canada. (2013). p. 28. http://etccdi.pacificclimate.org/software.shtml (accessed January 24, 2021).

[64] Brugnara Y Auchmann R Rutishauser T Gehrig R Pietragalla B Begert M. Homogeneity assessment of phenological records from the Swiss Phenology Network. Int J Biometeorol. (2020) 64:71–81. 10.1007/s00484-019-01794-y 31478107

[65] Kuglitsch FG Auchmann R Bleisch R Brönnimann S Martius O Stewart M. Break detection of annual Swiss temperature series. J Geophys Res Atmos. (2012) 117:D13105. 10.1029/2012JD017729

[66] Dahl Å Galán C Hajkova L Pauling A Sikoparija B Smith M. The onset, course and intensity of the pollen season. In: Sofiev M Bergmann K-C, editors. Allergenic Pollen: A Review of the Production, Release, Distribution and Health Impacts. Dordrecht: Springer Netherlands (2013). p. 29–70. 10.1007/978-94-007-4881-1_3

[67] Güsewell S Furrer R Gehrig R Pietragalla B. Changes in temperature sensitivity of spring phenology with recent climate warming in Switzerland are related to shifts of the preseason. Glob Change Biol. (2017) 23:5189–202. 10.1111/gcb.13781 28586135

[68] Estrella N Sparks TH Menzel A. Effects of temperature, phase type and timing, location, and human density on plant phenological responses in Europe. Clim Res. (2009) 39:235–48. 10.3354/cr00818

[69] Fu YH Zhao H Piao S Peaucelle M Peng S Zhou G. Declining globalwarming effects on the phenology of spring leaf unfolding. Nature. (2015) 526:104–7. 10.1038/nature15402 26416746

[70] Laube J Sparks TH Estrella N Höfler J Ankerst DP Menzel A. Chilling outweighs photoperiod in preventing precocious spring development. Glob Change Biol. (2013) 20:170–82. 10.1111/gcb.12360 24323535

[71] Peeters AG. Frost periods and beginning of the ash (*Fraxinus excelsior* L.) pollen season in Basel (Switzerland). Aerobiologia. (2000) 16:353–9. 10.1023/A:1026566625568

[72] Pauling A Gehrig R Clot B. Toward optimized temperature sum parameterizations for forecasting the start of the pollen season. Aerobiologia. (2014) 30:45–57. 10.1007/s10453-013-9308-0

[73] Newnham RM Sparks TH Skjøth CA Head K Adams-Groom B Smith M. Pollen season and climate: Is the timing of birch pollen release in the UK approaching its limit? Int J Biometeorol. (2013) 57:391–400. 10.1007/s00484-012-0563-5 22710742

[74] Wang H Wu C Ciais P Peñuelas J Dai J Fu Y. Overestimation of the effect of climatic warming on spring phenology due to misrepresentation of chilling. Nat Commun. (2020) 11:1–9. 10.1038/s41467-020-18743-8 33009378 PMC7532433

[75] Bossuyt N Wirthner J Dussoulier C Frund D Meisser M Kragten SA. Wann sollten intensiv genutzte Wiesengemäht werden? Agrarforschung Schweiz. (2018) 9:12–9. Available online at: https://www.agrarforschungschweiz.ch/artikel/2018_01_2353.pdf (accessed February 18, 2021).

[76] Anderegg WRL Abatzoglou JT Anderegg LDL Bielory L Kinney PL Ziska L. Anthropogenic climate change is worsening North American pollen seasons. Proc Natl Acad Sci. (2021) 118:e2013284118. 10.1073/pnas.2013284118 33558232 PMC7896283

[77] Buters J Prank M Sofiev M Pusch G Albertini R Annesi-Maesano I. Variation of the group 5 grass pollen allergen content of airborne pollen in relation to geographic location and time in season the HIALINE working group. J Allergy Clin Immunol. (2015) 136:87–95.e6. 10.1016/j.jaci.2015.01.049 25956508

[78] Pers-Kamczyc E Tyrała-Wierucka Z Rabska M Wronska-Pilarek D Kamczyc J. The higher availability of nutrients increases the production but decreases the quality of pollen grains in Juniperus communis L. J Plant Physiol. (2020) 248:153156. 10.1016/j.jplph.2020.153156 32244105

[79] García-Mozo H Galán C Alcázar P De La Guardia CD Nieto-Lugilde D Recio M. Trends in grass pollen season in southern Spain. Aerobiologia. (2010) 26:157–69. 10.1007/s10453-009-9153-3

[80] Baselland S. Raum und Umwelt. Available online at: https://www.statistik.bl.ch/web_portal/2_5_2?year=1982 (accessed January 03, 2021).

[81] Emberlin J Mullins J Corden J Jones S Millington W Brooke M. Regional variations in grass pollen season in the UK, long-term trends and forecast models. Clin Exp Allergy. (1999) 29:347–56. 10.1046/j.1365-2222.1999.00369.x 10202342

[82] González Minero FJ Candau P Tomás C Morales J. Airborne grass (Poaceae) pollen in southern Spain. Results of a 10-year study (1987–96). Allergy. (1998) 53:266–74. 10.1111/j.1398-9995.1998.tb03886.x 9542606

[83] Gehrig R. The influence of the hot and dry summer 2003 on the pollen season in Switzerland. Aerobiologia. (2006) 22:27–34. 10.1007/s10453-005-9013-8

[84] Aguila E Auer I Brunet M Peterson TC Wieringa J. Guidelines on Climate Metadata and Homogenization. (2003). Available online at: https://www.wmo.int/pages/prog/wcp/wcdmp/documents/WCDMP-53.pdf (accessed February 18, 2021).

[85] Saar M Meltsov V. Passports of sampling sites in routine aerobiological monitoring of outdoor air. Aerobiol Monogr. (2011) 1:215–31.

[86] Oteros J Buters J Laven G Röseler S Wachter R Schmidt-Weber C. Errors in determining the flow rate of Hirst-type pollen traps. Aerobiologia. (2017) 33:201–10. 10.1007/s10453-016-9467-x

[87] Clot B Gilge S Hajkova L Magyar D Scheifinger H Sofiev M. The EUMETNET AutoPollen programme: establishing a prototype automatic pollen monitoring network in Europe. Aerobiologia. (2020) 1–9. 10.1007/s10453-020-09666-433169045

