# Supplementary material for: 50 Years of Pollen Monitoring in Basel (Switzerland) Demonstrate the Influence of Climate Change on Airborne Pollen
Source: Front Allergy. 2021 May 28;2:677159. doi: 10.3389/falgy.2021.677159 (PMC8974697; doi:10.3389/falgy.2021.677159)

Supplementary Material

1 Supplementary Tables

Supplementary Table S1. Pollen season characteristics and pollen trends 1969-2018. Significant trends are marked in bold ($p \leq 0.05$). The values for start and end dates and duration are calculated by averaging the dates for all definitions per year and from this data series the mean, minimum and maximum is calculated. Trends for mean start and end dates and the duration are calculated as well for the yearly mean of the 8 definitions.

**Pollen taxa**		**Start [date]**	**End [date]**	**Duration [days]**	**APIn [pollen*day/m3**	**Peak value [pollen/m3]**	**# high days (≥50 p/m3]**
Corylus	Mean	03.02.	16.03.	43	1527	259	8
	Min	05.01.	23.02.	23	285	44	0
	Max	13.03.	09.04.	73	4526	1100	22
	trend abs	**-22.5**	-5.6	**17.4**	**1242.5**	**159.3**	**8.2**
	p-value	**0.020**	0.668	**0.017**	**0.001**	**0.023**	**0.001**
Alnus	Mean	11.02.	24.03.	43	2801	527	12
	Min	08.01.	28.02.	22	320	35	0
	Max	10.03.	18.04.	74	8588	2550	28
	trend abs	-11.6	-12.5	-2.4	**2407.5**	**389.8**	**13.6**
	p-value	0.202	0.184	0.722	**0.003**	**0.010**	**0.001**
Taxus/Cupressaceae	Mean	12.02.	27.04.	75	12525	2539	31
	Min	09.01.	10.04.	43	3975	340	14
	Max	16.03.	15.05.	112	28116	10000	63
	trend abs	**-27.7**	-5.6	**21.8**	**10272.7**	**1379.1**	**18.4**
	p-value	**0.005**	0.132	**0.022**	**<0.001**	**0.037**	**0.001**
Fraxinus	Mean	27.03.	02.05.	37	3637	626	14
	Min	27.02.	08.04.	17	485	79	3
	Max	16.04.	24.05.	65	12632	2828	25
	trend abs	**-18.4**	**-30.6**	**-15.0**	**2942.0**	**497.5**	**6.8**
	p-value	**0.004**	**<0.001**	**0.002**	**0.002**	**0.003**	**0.015**
Betula	Mean	31.03.	05.05.	36	6015	1010	19
	Min	12.03.	15.04.	21	1150	182	3
	Max	17.04.	28.05.	60	14596	3040	37
	trend abs	**-9.9**	**-13.4**	-3.7	989.0	8.2	4.9
	p-value	**0.037**	**0.001**	0.447	0.498	0.980	0.086
Carpinus	Mean	29.03.	25.04.	28	1616	336	7
	Min	16.02.	03.04.	10	14	5	0
	Max	20.04.	17.05.	57	9794	2340	18
	trend abs	-10.3	**-13.9**	-2.9	**1215.2**	**251.1**	**4.5**
	p-value	0.065	**0.004**	0.651	**0.020**	**0.015**	0.089
Platanus	Mean	10.04.	07.05.	28	1653	314	8
	Min	05.03.	15.04.	12	235	55	1
	Max	03.05.	24.05.	57	3682	1060	14
	trend abs	-6.9	**-14.4**	-5.4	**1544.7**	**212.3**	**5.6**
	p-value	0.204	**<0.001**	0.277	**<0.001**	**0.003**	**0.001**

Fagus	Mean	14.04.	20.05.	37	1678	232	10
	Min	12.02.	07.05.	14	22	4	0
	Max	06.05.	24.06.	105	6043	1031	23
	trend abs	**-16.4**	-7.1	9.1	1290.3	155.9	**9.8**
	p-value	**0.002**	0.064	0.179	0.139	0.181	**0.045**
Quercus	Mean	18.04.	25.05.	37	3226	418	16
	Min	28.03.	10.05.	24	883	86	2
	Max	06.05.	09.06.	57	9148	1565	28
	trend abs	**-19.3**	**-16.3**	2.6	**2048.7**	**291.4**	**7.5**
	p-value	**<0.001**	**<0.001**	0.530	**0.002**	**0.002**	**0.020**
Pinus	Mean	03.05.	14.06.	43	3490	467	18
	Min	11.04.	21.05.	21	1349	100	8
	Max	23.05.	01.08.	83	14562	2244	32
	trend abs	**-23.7**	**-9.8**	**12.0**	1119.2	122.5	4.3
	p-value	**<0.001**	**0.037**	**0.036**	0.105	0.262	0.108
Poaceae	Mean	09.05.	12.08.	96	6657	458	38
	Min	21.04.	16.07.	67	3319	156	16
	Max	27.05.	15.09.	127	14445	1316	67
	trend abs	-6.1	**-26.0**	**-18.4**	-408.3	-119.4	-1.3
	p-value	0.061	**<0.001**	**0.004**	0.694	0.219	0.682
Urticaceae	Mean	02.06.	07.09.	99	6104	243	43
	Min	03.05.	11.08.	71	1310	56	1
	Max	20.06.	28.09.	131	13935	500	76
	trend abs	**-14.3**	-2.6	**12.3**	**3564.0**	**122.5**	**20.6**
	p-value	**<0.001**	0.403	**0.014**	**0.005**	**0.027**	**0.018**
Populus	Mean				897	157	5
	Min				155	25	0
	Max				2870	614	17
	trend abs				**998.6**	**183.8**	**6.3**
	p-value				**<0.001**	**<0.001**	**<0.001**
Picea	Mean				763	152	3
	Min				4	1	0
	Max				3995	1000	15
	trend abs				380.7	**98.0**	0.0
	p-value				0.051	**0.046**	0.078
		**Start [date]**	**End [date]**	**Duration [days]**	**APIn [pollen*day/m3**	**peak value [pollen/m3]**	**# high days (≥15p/m3]**
Rumex	Mean				645	42	14
	Min				143	11	0
	Max				1720	130	49
	trend abs				**-482.5**	**-25.2**	**-19.6**
	p-value				**<0.001**	**0.001**	**<0.001**
Plantago	Mean				843	35	18
	Min				321	11	0
	Max				2030	85	57
	trend abs				**-551.3**	**-31.0**	**-23.5**
	p-value				**<0.001**	**<0.001**	**<0.001**

Artemisia	Mean				161	21	2
	Min				60	6	0
	Max				475	88	10
	trend abs				**-147.0**	**-13.5**	**-4.1**
	p-value				**<0.001**	**<0.001**	**<0.001**
Ambrosia	Mean				75	14	1
	Min				10	2	0
	Max				270	60	5
	trend abs				-29.1	0.0	0.0
	p-value				0.171	0.972	0.379

Supplementary Table S2. Correlation coefficients of different pollen season definitions of the Poaceae start dates (A) and end dates (B). * p-value ≤ 0.05, ** p-value ≤ 0.01, *** p-value ≤ 0.001.

**A: Poaceae start dates of MPS**								
**definition**	**clin_bet**	**clin_poa**	**perc95**	**perc90**	**tr20**	**tr30**	**3d20**	**moving**
**clin_bet**	1.000							
**clin_poa**	0.432**	1.000						
**perc95**	0.513***	0.714***	1.000					
**perc90**	0.734***	0.584***	0.876***	1.000				
**tr20**	0.357*	0.513***	0.849***	0.692***	1.000			
**tr30**	0.913***	0.440***	0.524***	0.680***	0.369**	1.000		
**3d20**	0.877***	0.453***	0.472***	0.698***	0.310*	0.826***	1.000	
**moving**	0.812***	0.603***	0.762***	0.821***	0.663***	0.794***	0.755***	1.000

**B: Poaceae end dates of MPS**								
**definition**	**clin_bet**	**clin_poa**	**perc95**	**perc90**	**tr20**	**tr30**	**3d20**	**moving**
**clin_bet**	1.000							
**clin_poa**	0.542***	1.000						
**perc95**	0.392**	0.677***	1.000					
**perc90**	0.551***	0.685***	0.937***	1.000				
**tr20**	0.787***	0.608***	0.472***	0.624***	1.000			
**tr30**	0.806***	0.604***	0.460***	0.596***	0.854***	1.000		
**3d20**	0.837***	0.461***	0.309*	0.442***	0.659***	0.697***	1.000	
**moving**	0.818***	0.561***	0.382**	0.497***	0.721***	0.693***	0.724***	1.000

Supplementary Table S3. Correlation coefficients of different pollen season definitions of the *Betula* start dates (A) and end dates (B). * p-value ≤ 0.05, ** p-value ≤ 0.01, *** p-value ≤ 0.001.

**A *Betula* start dates of MPS**								
**definition**	**clin_bet**	**clin_poa**	**perc95**	**perc90**	**tr20**	**tr30**	**3d20**	**moving**
**clin_bet**	1.000							
**clin_poa**	0.954***	1.000						
**perc95**	0.952***	0.938***	1.000					
**perc90**	0.971***	0.951***	0.970***	1.000				
**tr20**	0.958***	0.952***	0.960***	0.952***	1.000			
**tr30**	0.955***	0.953***	0.960***	0.950***	0.994***	1.000		
**3d20**	0.977***	0.942***	0.925***	0.972***	0.936***	0.935***	1.000	
**moving**	0.962***	0.960***	0.966***	0.955***	0.994***	0.993***	0.943***	1.000

**B *Betula* end dates of MPS**								
**definition**	**clin_bet**	**clin_poa**	**perc95**	**perc90**	**tr20**	**tr30**	**3d20**	**moving**
**clin_bet**	1.000							
**clin_poa**	0.644***	1.000						
**perc95**	0.460***	0.582***	1.000					
**perc90**	0.647***	0.572***	0.849***	1.000				
**tr20**	0.652***	0.869***	0.455***	0.474***	1.000			
**tr30**	0.872***	0.612***	0.455***	0.622***	0.610***	1.000		
**3d20**	0.866***	0.538***	0.399**	0.647***	0.576***	0.773***	1.000	
**moving**	0.859***	0.763***	0.522***	0.660***	0.702***	0.923***	0.727***	1.000

Supplementary Table S4. Correlation coefficient between APIn and temperature during pre-season and main pollen season (MPS). Only significant correlations with $p \leq 0.05$ are shown. Significant correlations outside pre-season and MPS are not shown.

	Jan	Mar	Apr	May	Jun	Jul	JanFeb	JanMar	JanApr	JanJun	FebApr	FebMay	FebJun	MarApr	MarMay	MarJun	MarJul	MarAug
Corylus	0.354		0.208				0.356	0.362	0.451		0.385			0.357				
Alnus																		
Taxus/Cupr			0.298						0.334		0.396			0.408				
Fraxinus			0.184															
Betula																		
Carpinus							0.241											
Populus		0.263	0.478					0.23	0.355		0.367			0.551				
Platanus			0.441						0.244		0.319			0.466				
Fagus									0.28			0.334		0.177	0.239			
Quercus			0.396	0.294								0.32		0.314	0.354			
Pinus		0.399		0.343						0.32		0.344	0.322		0.411			
Picea				0.368						0.331	0.401	0.452	0.43		0.427	0.351		
Poaceae							0.421	0.404										
Urticaceae																		
Plantago			-0.418		-0.472					-0.356			-0.404	-0.354	-0.375	-0.441	-0.464	-0.426
Rumex			-0.336		-0.411													
Artemisia			-0.554		-0.571	-0.426				-0.458	-0.332	-0.37	-0.475	-0.464	-0.442	-0.527	-0.604	-0.582
Ambrosia			-0.329															

	AprMay	AprJun	AprJul	AprAug	MayJun	MayJul	MayAug	JunJul	JunAug	JunSep
Corylus										
Alnus										
Taxus/Cupr										
Fraxinus										
Betula										
Carpinus										
Populus										
Platanus										
Fagus										
Quercus										
Pinus										
Picea	0.414	0.301								
Poaceae										
Urticaceae										
Plantago	-0.391	-0.483	-0.508	-0.478	-0.386	-0.473		-0.457		
Rumex		-0.356	-0.392					-0.389		
Artemisia	-0.446	-0.568	-0.63	-0.628	-0.45	-0.585	-0.556	-0.617	-0.574	-0.551

2 Supplementary Figures

Supplementary Figure S1. Comparison of phenological observations of the full flowering of *Corylus* and the start of pollen measurement in Basel 1969-2018. The goal was to check if the start of the *Corylus* (and *Alnus*) pollen season was missed in years with late start of pollen measurements. Phenological observations were available for the stations of Basel Binningen (distance to the pollen trap 2 km), Reinach (7 km), Therwil (7 km), Liestal (14 km). The graph shows the doy (day of year) of the averaged phenology date of full flowering of *Corylus* of the four stations (missing data are occurring in the phenology data series) and the yearly doy of the start of pollen measurement. In the years, 1969, 1974-1976, 1978-1979, 1982 pollen measurement started after the observed full flowering of *Corylus*. These years were omitted for the calculation of the *Corylus* and *Alnus* pollens season start dates.

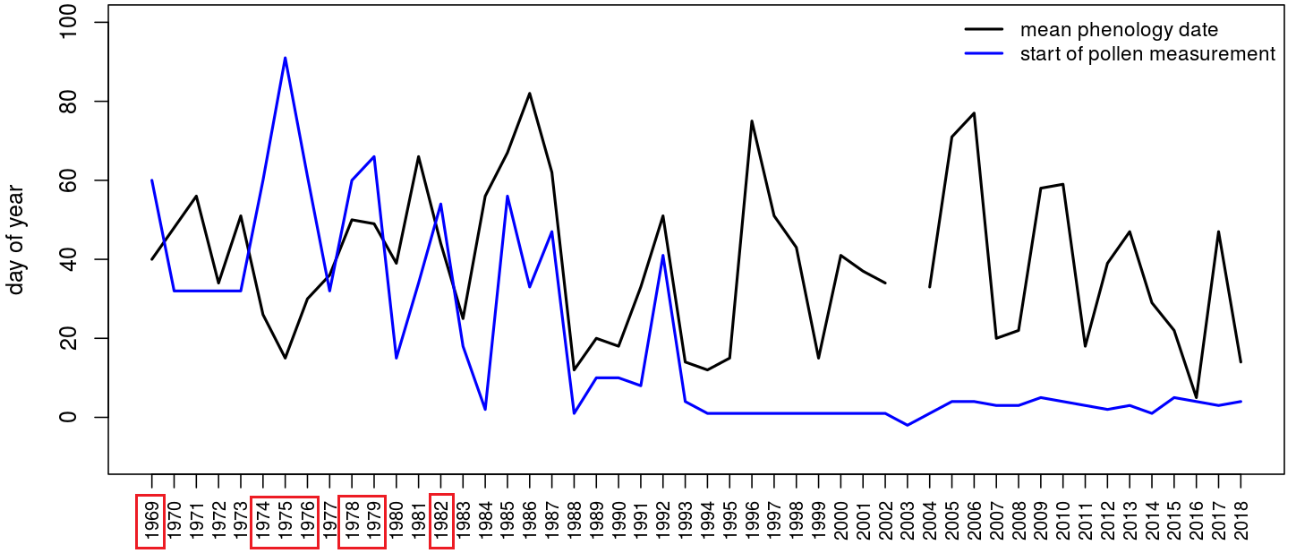

Supplementary Figure S2. Breakpoint detection of APIn data series 1969-2018 in Basel with the method of Penalized Maximal F test (PMF). Pollen data series with a significant breakpoint (A) and not significant breakpoint (B).

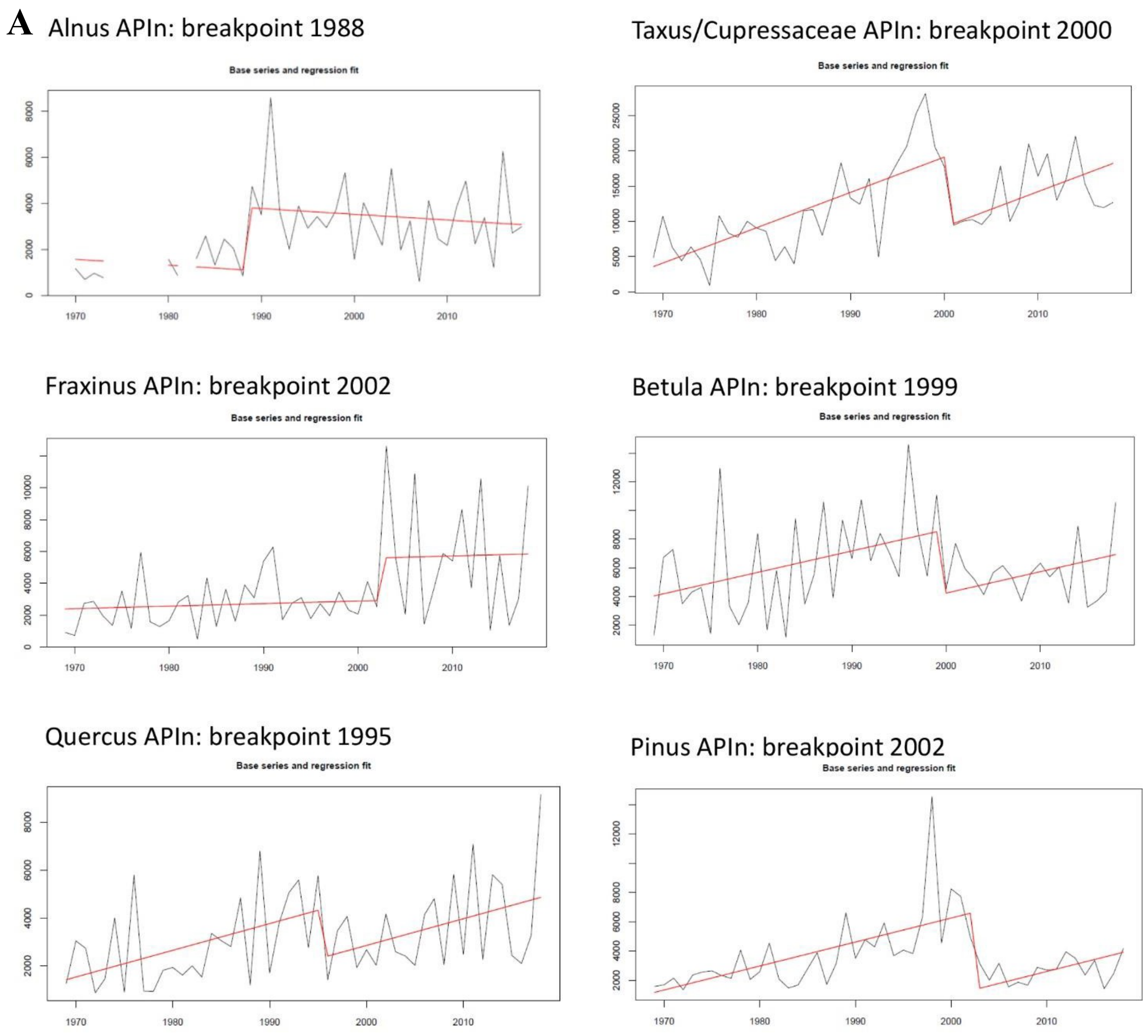

Picea APIn: breakpoint 2003

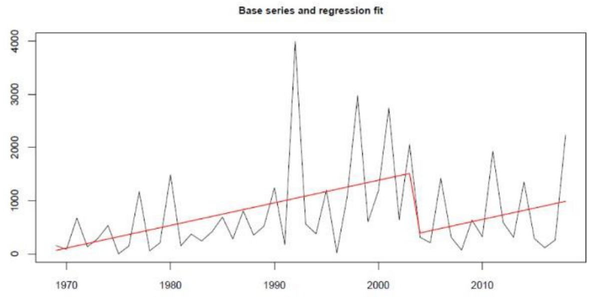

Poaceae APIn: breakpoint 2001

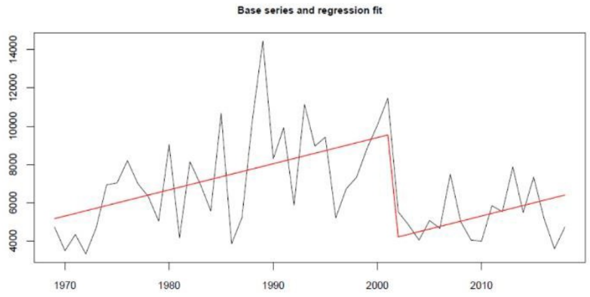

Urticaceae APIn: breakpoint 1994

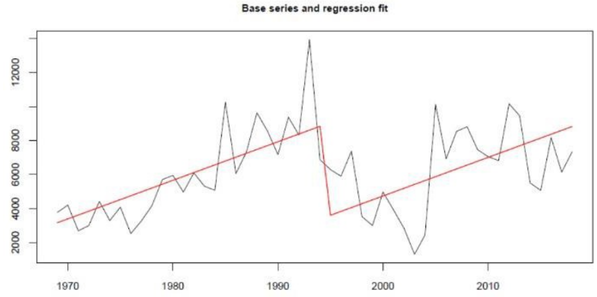

Plantago APIn: breakpoint 1984

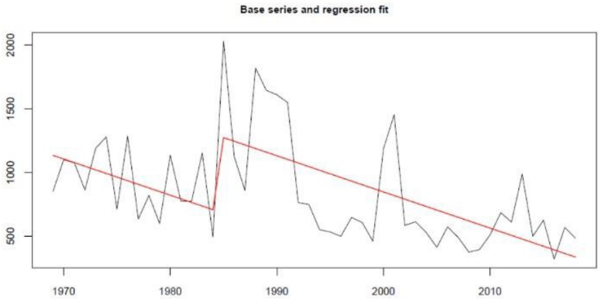

Rumex APIn: breakpoint 1986

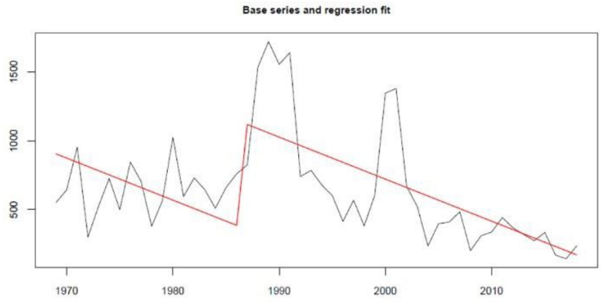

Ambrosia APIn: breakpoint 1987

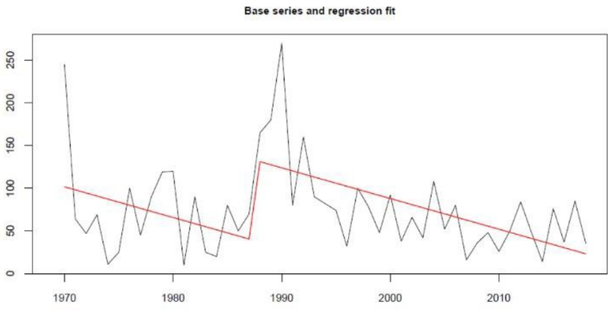

B

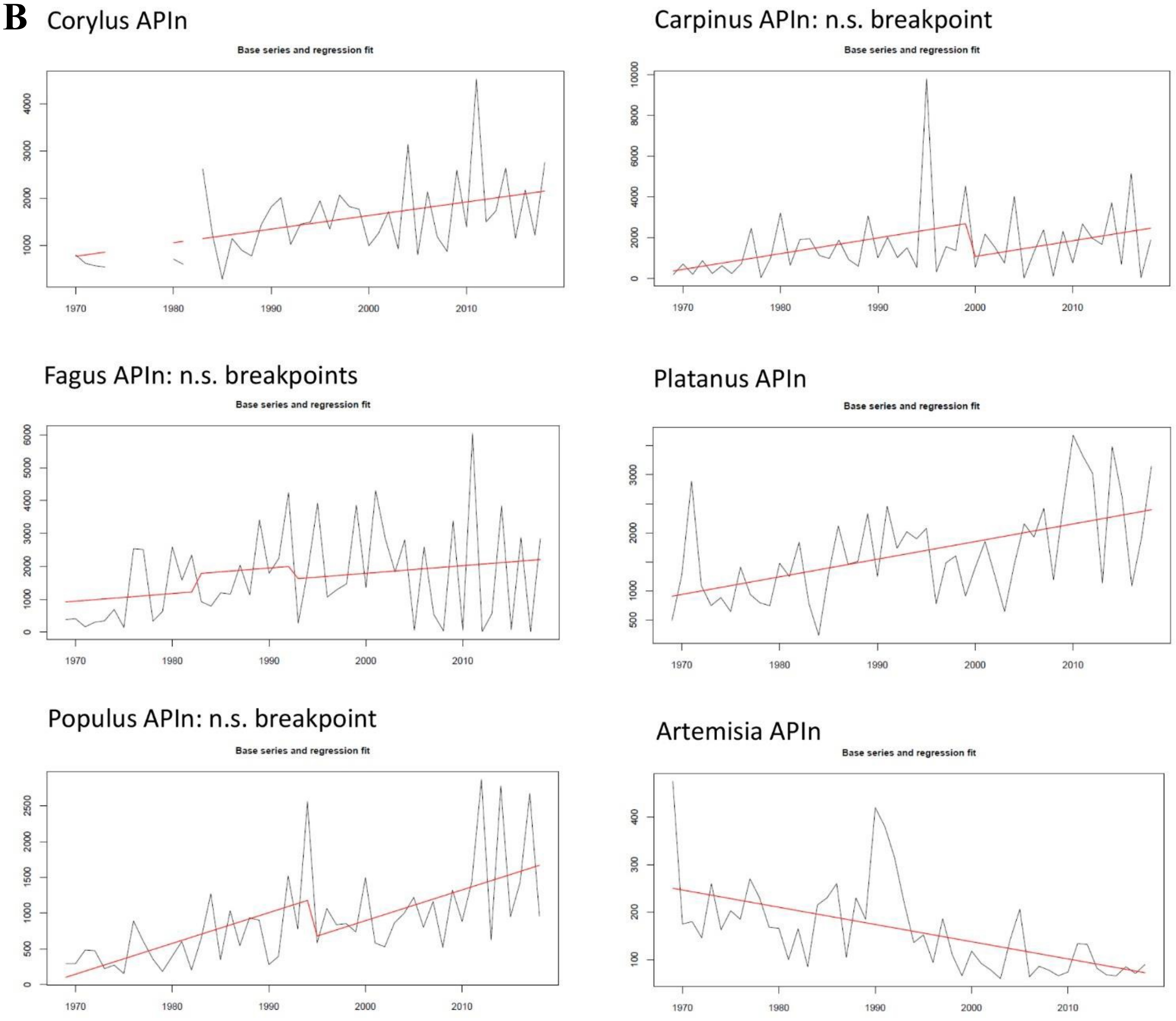
Corylus APIn
Base series and regression fit
Carpinus APIn: n.s. breakpoint
Base series and regression fit
Fagus APIn: n.s. breakpoints
Base series and regression fit
Platanus APIn
Base series and regression fit
Populus APIn: n.s. breakpoint
Base series and regression fit
Artemisia APIn
Base series and regression fit

Supplementary Figure S3. Absolute trends [days] for the 50-year period 1969-2018 of the start, end and duration of the pollen season for herbaceous taxa with pollen season definitions with low thresholds. Red points show significant trends (p-value ≤ 0.05), black points show non-significant trends. The pollen season definitions are mostly different from the ones used for all other pollen taxa, because threshold definitions fail in many years to define a pollen season of these herbaceous taxa. "clin_low": 1st day of 5 days with ≥1 pollen/m^3 (out of 7 consecutive days) and sum of 5 days ≥10 pollen/m3. "3d1" and "5d1": three and five consecutive days with at least 1 pollen/m^3. "moving", "clin_poa" and the percentage definitions belong to the eight standard definitions.

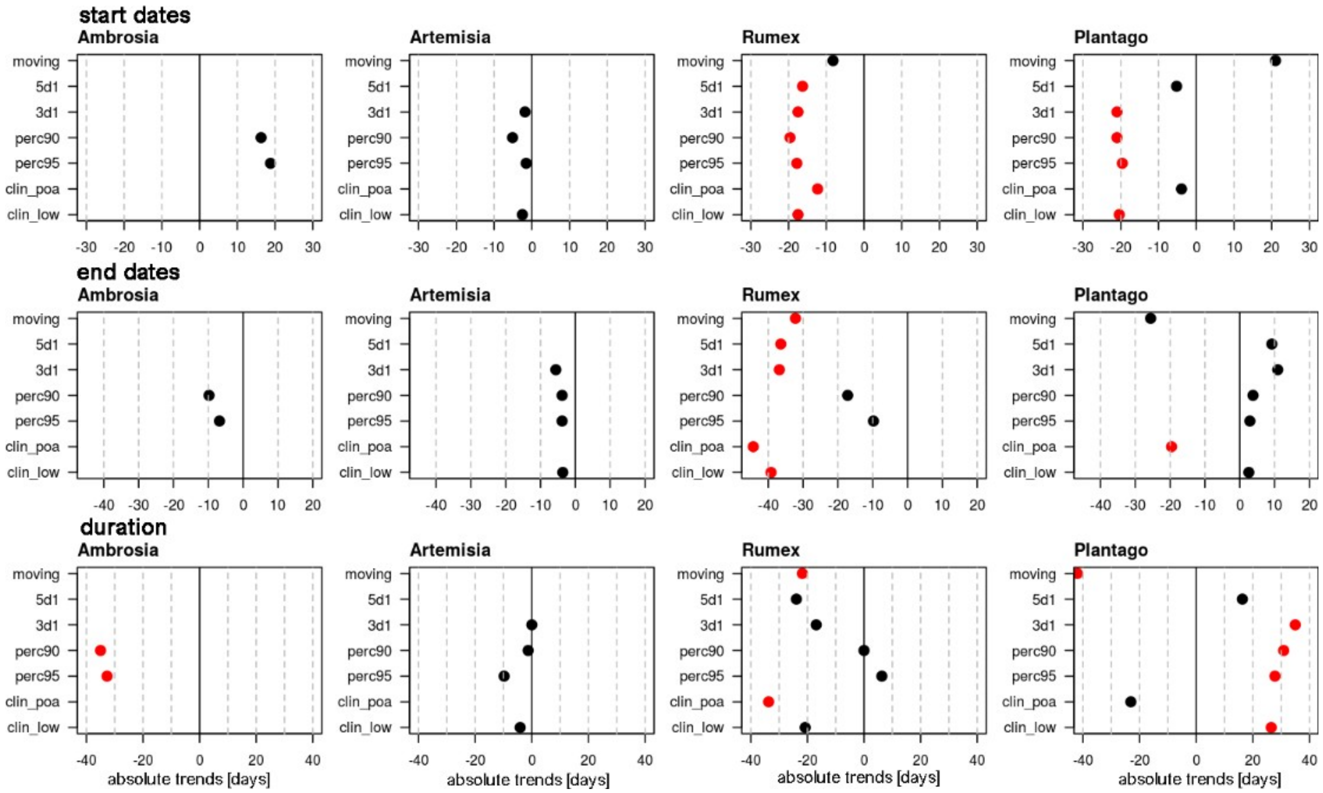

Supplementary Figure S4. Linear trends of the peak value (pollen/m^3) for the 50-year period 1969-2018. Red trend lines show significant trends (p-value ≤ 0.05), black trend lines show non-significant trends.

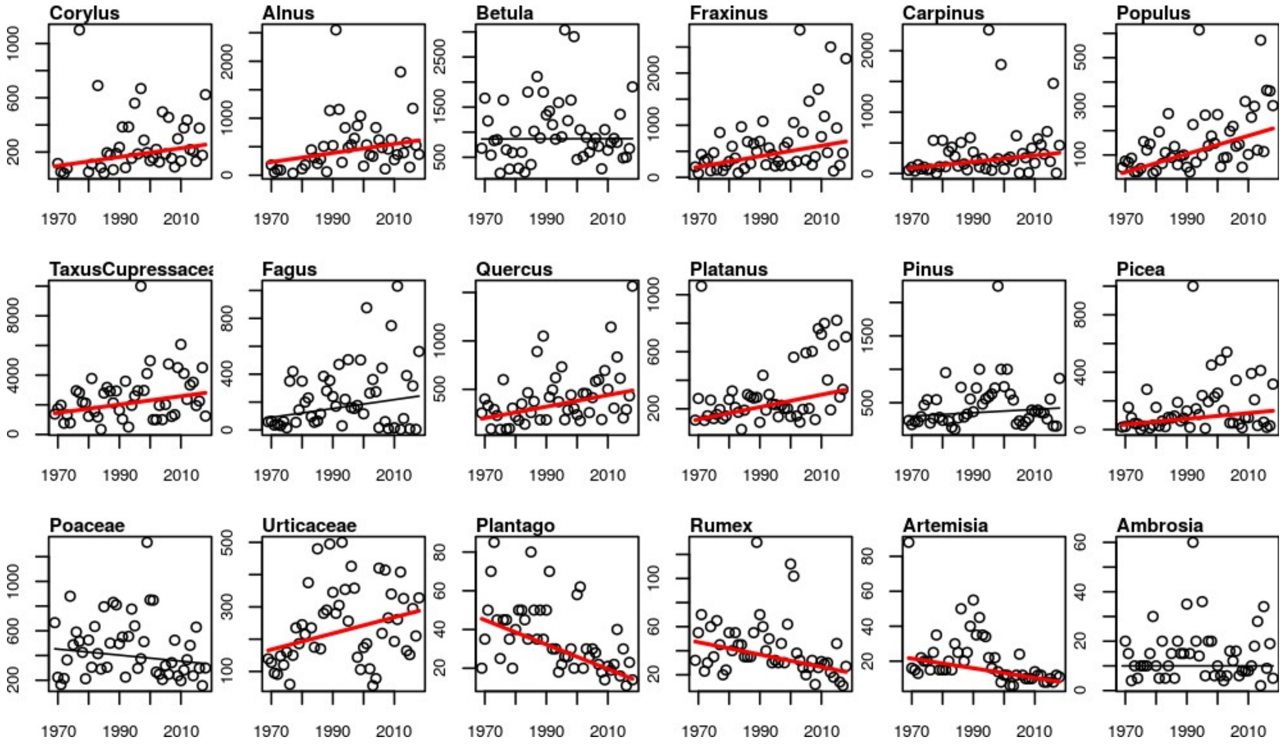

Supplementary Figure S5. Linear trends of the number of high pollen days (days ≥ 50 pollen/m^3) for the 50-year period 1969-2018. Red trend lines show significant trends (p-value ≤ 0.05), black trend lines show non-significant trends. The herbaceous taxa *Plantago, Rumex, Artemisia* and *Ambrosia* do normally not reach the threshold of 50 pollen/m^3.

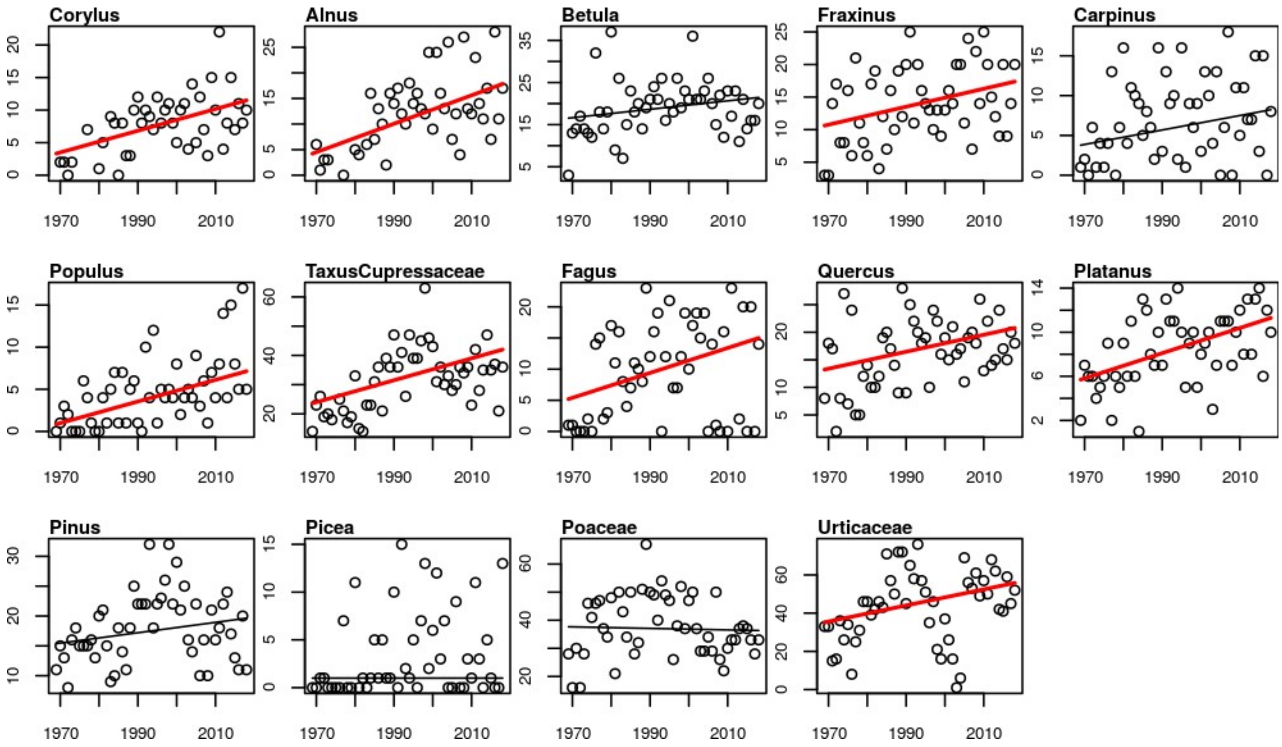

Supplementary Figure S6. *Betula* pollen start dates with eight different definitions against mean March temperature with a LOWESS smoothed line for showing the relationship for Basel 1969-2018. The smoothed line is nearly linear for the whole range of March temperatures. A: smoother span f=0.6, R function “lowess”, B: smoother span f=0.5, R function “lowess”.

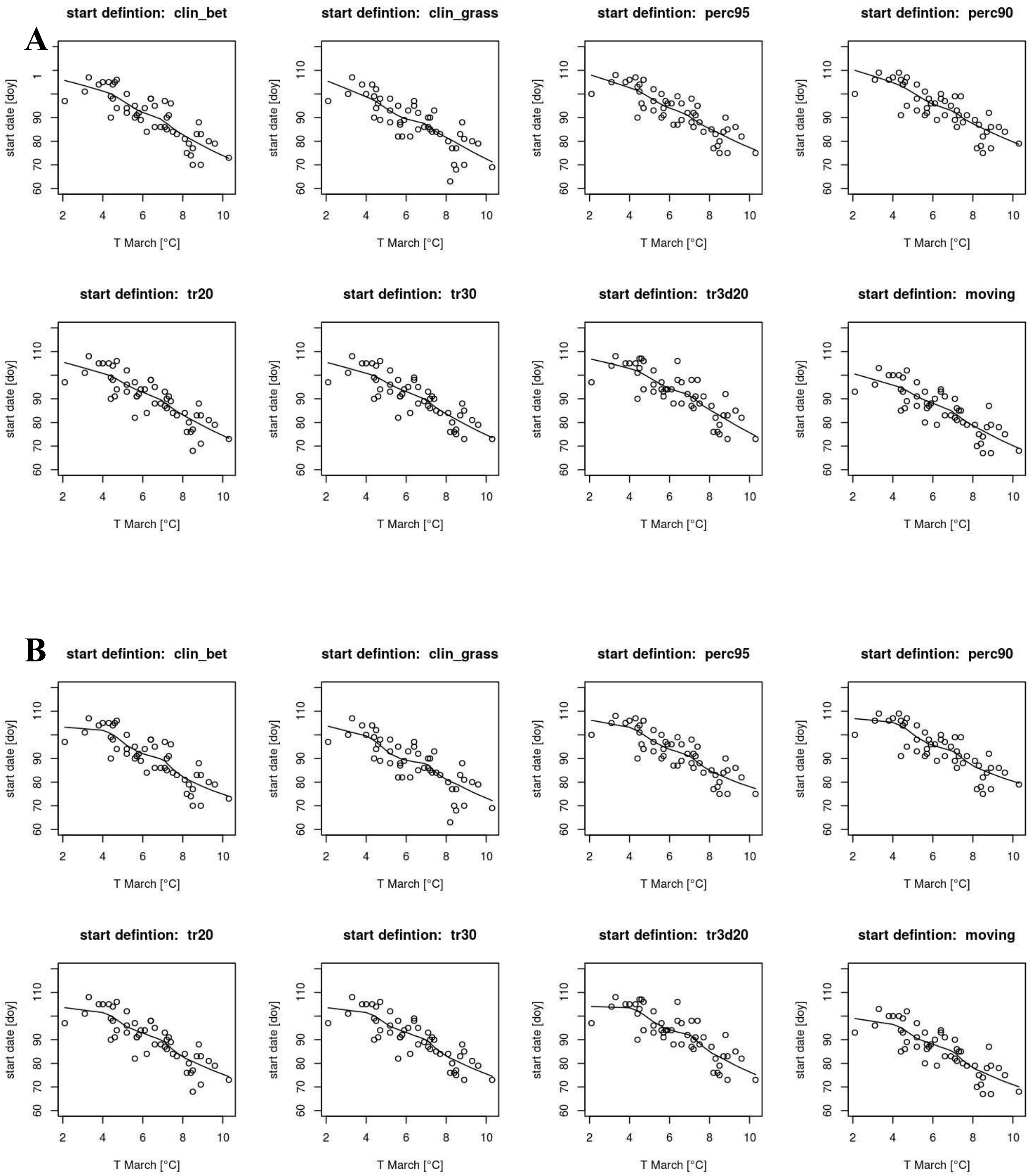

Supplement: Supplementary file 1 [file Data_Sheet_1.pdf]
